# Aminosulfonated Graphene as a Catalyst for Efficient Production of Biodiesel from Fatty Acids and Crude Vegetable Oils

**DOI:** 10.1002/cssc.202402488

**Published:** 2025-04-18

**Authors:** Aby Cheruvathoor Poulose, Hugo Bares, Dagmar Zaoralová, Ivan Dědek, Michal Otyepka, Aristides Bakandritsos, Radek Zbořil

**Affiliations:** ^1^ Regional Centre of Advanced Technologies and MaterialsCzech Advanced Technology and Research Institute (CATRIN) Palacký University in Olomouc Šlechtitelů 27 78371 Olomouc Czech; ^2^ Present address: LEPTY 14 avenue Pey‐Berland 33600 Pessac France; ^3^ IT4InnovationsVŠB−Technical University of Ostrava 17. listopadu 2172/15, Ostrava 708 00 Poruba Czech; ^4^ Nanotechnology Centre Centre for Energy and Environmental Technologies VŠB–Technical University of Ostrava 17. listopadu 2172/15, Poruba 708 00 Ostrava Czech

**Keywords:** biodiesel, esterification, graphene catalysts, heterogeneous acid catalysts, solid acid catalysis, transesterification

## Abstract

Climate change and the depletion of fossil fuels increase the demand for sustainable energy. Biodiesel synthesized using heterogeneous acid catalysts is a promising clean‐energy carrier that supports a circular carbon economy. Herein, the efficient synthesis of biodiesel is reported using a reusable solid acid graphene catalyst functionalized with a natural aminosulfonic acid. Experimental and theoretical studies reveal the key role of functionalities that simultaneously contain amino and sulfonate groups, which impart superior acidity. Excellent activity and selectivity for oleic acid conversion to oleic acid methyl esters (a sustainable biofuel) are obtained, offering a strategy for achieving improved catalytic performance compared to earlier or benchmark catalysts in the field. Notably, the catalyst also effectively converts common vegetable oils into biodiesel via transesterification and facilitates carbohydrate dehydration to value‐added chemicals, demonstrating broad applicability. Two additional variants of aminosulfonic acid‐functionalized graphene show similar activity, confirming the crucial role of these functionalities in achieving high acidity and catalytic performance. The development of such potent, recyclable catalysts is crucial because acid catalysis is highly versatile, underpinning many biological and synthetic transformations.

## Graphical abstract

The covalent integration of amino and sulfonate groups on the graphene surface significantly enhances activity in acid‐catalyzed reactions, such as in biodiesel production and dehydration of saccharides. Developing such heterogeneous, recyclable catalysts is vital, as acid catalysis represents one of the most general and versatile strategies in organic synthesis, underpinning a wide range of biological and chemical transformations.

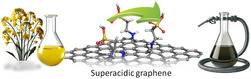

## 1 Introduction

The escalating impact of climate change and the depletion of fossil fuel reserves is driving an increased demand for sustainable and clean energy.^[^ ^1^, ^2^, ^3^, ^4^, ^5^ ^]^ Biodiesel is an eco‐friendly renewable energy carrier that effectively operates in existing automobile engines.^[^ ^6^, ^7^ ^]^ Its properties closely match those of fossil diesel; however, it exhibits a higher flash point, improved lubricity, and lower emissions due to its higher oxygen content.

Biodiesel is primarily synthesized by esterification of free fatty acids or transesterification of long‐chain triglycerides with alcohols, employing acid or base catalysts. In industrial processes, homogeneous base catalysts like NaOH and KOH are extensively utilized for transesterification, while homogeneous acid catalysts, such as HCl, H_2_SO_4_, and H_3_PO_4_, are widely employed due to their high activity at low temperatures. When crude bio‐oils contain high levels of fatty acids, the activity of base catalysts is compromised, leading to saponification and reduced biodiesel quality. Conversely, acid catalysts exhibit versatility by facilitating both triglyceride transesterification and fatty acid esterification with alcohols, even in the presence of water.^[^ ^8^ ^]^ Esterification reactions are important not only in biodiesel synthesis but also for the production of a large variety of chemicals and pharmaceuticals,^[^ ^9^, ^10^ ^]^ with sulfuric acid remaining the industrial catalyst of choice.

The development of heterogeneous solid acid catalysts is of vital importance for industrial catalysis^[^ ^11^ ^]^ due to their facile separation, improved environmental impact, and avoidance of equipment corrosion, a common pitfall with homogeneous acid catalysts. A variety of heterogeneous acid catalysts have been developed, such as metal oxides, hydrotalcites, ion‐exchange resins, basic oxides, sulfonic acid‐functionalized carbons, and other supports.^[^ ^9^, ^12^, ^13^, ^14^, ^15^ ^]^ To evaluate their practical performance, especially given their structural diversity, it is beneficial to utilize specific productivity (SP, i.e., the moles of product formed per total catalyst mass per hour). SP complements the turnover frequency (TOF; which accounts for the activity with respect to the active sites only). For instance, Toda et al. reported an SP = 12.5 mmol g^−^^1^ h^−^^1^ in the esterification of oleic acid at 80 °C for 4 h using sulfonated carbon derived from sugars.^[^ ^13^ ^]^ Resorcinol/formaldehyde‐derived sulfonated carbons resulted in 22 mmol g^−^^1^ h^−^^1^ (60 °C, 6 h).^[^ ^14^ ^]^ Polystyrene sulfonic acid resins in hollow silica, or wood‐derived sulfonated carbons achieved less than 13 mmol g^−^^1^ h^−^^1^.^[^ ^9^, ^15^ ^]^ In general, SP values approaching 40 mmol g^−^^1^ h^−^^1^ have been rarely reported (Table S3, Supporting Information). The synthesis of these catalysts, in most cases, requires harsh conditions involving hazardous chemicals (such as concentrated H_2_SO_4_, fuming SO_3_, or chlorosulfonic acid) under high temperatures or pressures (200–600 °C and 1–10 MPa). Moreover, improving the catalytic activity at low temperatures will substantially promote their industrial applicability.^[^ ^16^, ^17^ ^]^

**Figure 1:**
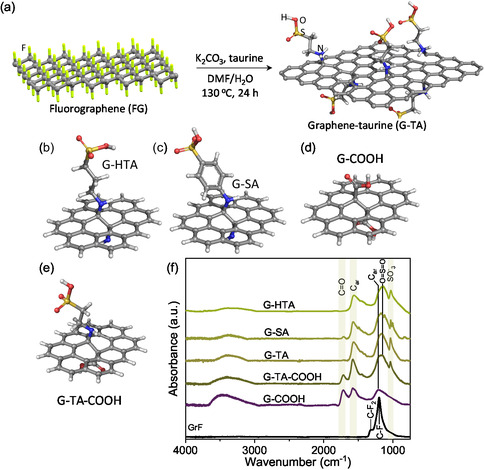
a–e) Synthetic procedure and finite models of the starting FG and of the functionalized graphene derivatives synthesized in this work with various molecules ascribing acidic properties. Carbon atoms are gray, nitrogen blue, oxygen red, sulfur yellow, and hydrogen white. f) FTIR spectra of the corresponding materials.

Recently, we reported a super‐acidic and covalently functionalized graphene derivative with a natural amino acid (taurine or 2‐aminoethane sulfonic acid, **Figure** **1**a) displaying high efficiency as a heterogeneous acid catalyst for glycerol acetylation.^[^ ^18^ ^]^ Encouraged by these findings, we anticipated its versatility across various acid‐catalyzed reactions. Herein, we report on the efficient and fast biodiesel production at low temperatures utilizing this potent acidic graphene catalyst (G‐TA) and we also verify the key role of the aminosulfonic acid functionalities for obtaining high catalytic activity by testing two more types of aminosulfonated graphenes. The catalyst substantially outperformed state‐of‐the‐art solid‐state acid catalysts, as detailed later, delivering remarkable activity and selectivity for oleic acid conversion. Moreover, we demonstrate its broad applicability and potency in other acid‐catalyzed reactions, including the transesterification of vegetable oils and the dehydration of carbohydrates to yield value‐added chemicals. The development of recyclable superacid catalysts is crucial in the industry, as acid catalysis is arguably the most general and versatile approach for organic reactions, ubiquitous in an extraordinary range of biological and synthetic transformations.^[^ ^11^, ^16^, ^17^ ^]^

## 2 Experimental Section

### 2.1 Materials and Reagents

Graphite fluorinated polymer (or graphite fluoride, GrF, >61 mass % in F), 2–aminoethanesulfonic acid (taurine, 99.5%), 3‐amino‐1‐propanesulfonic acid (homotaurine 99%), sulfanilic acid (99%), potassium carbonate, N,N‐dimethylformamide (DMF, 99.8%), acetone (p.a.), ethanol (p.a.), methanol (99.9%), oleic acid, palmitic acid, stearic acid, triacetin, tributyrin, triolein, sulfuric acid, ethyl acetate, soybean oil, cotton seed oil, castor oil, rapeseed oil, fructose, and xylose were obtained from Sigma‐Merck. Ultrapure water was used (conductivity ≤ 0.5 μS cm^−1^).

### 2.2 Catalyst Synthesis

The taurine‐functionalized graphene (G–TA) catalyst was prepared according to our previous report.^[^ ^18^ ^]^ In a typical procedure, 1 g of GrF (32 mmol based on C‐F units) was dispersed in 48 mL of DMF in a round‐bottom flask, stirred for 3 days, and then sonicated (Bandelin Sonorex DT 255 H, frequency 35 kHz, power 640 W, effective power 160 W) for 4 h to exfoliate GrF into fluorographene (FG). The samples were manually shaken every 20 min to homogenize the sedimented solid in the dispersion. Subsequently, the dispersion was stirred at room temperature for an additional 24 h. Taurine (4 g, 32 mmol) and K_2_CO_3_ (5.3 g, a 1.2 molar excess relative to taurine) were separately dissolved in 6 mL of ultrapure water. The GrF dispersion in DMF was combined with the K_2_CO_3_ solution in a round‐bottom flask and then added to the taurine solution, ensuring basic conditions to maintain the amino group of taurine deprotonated and nucleophilic. The mixture was heated at 130 °C under magnetic stirring (300 rpm) for 24 h in an oil bath with a water‐cooled reflux condenser. After cooling to room temperature, the resulting solid was isolated by centrifugation at 20 000 rcf for 8 min and washed sequentially with various solvents to remove impurities: twice with hot DMF, once with DMF, once with hot acetone, twice with acetone, three times with ethanol, twice with ultrapure water, twice with 2% HCl, and three times with ultrapure water, until the conductivity of the wash water dropped below 200 μS cm^−1^. The precipitate was redispersed in ultrapure water and purified by transferring it into a cellulose dialysis membrane (Visking, molecular weight cut‐off 14 kDa). The sealed membrane was placed in a large beaker (capacity > 5 L) filled with distilled water, and the external water in the beaker was replaced twice daily. The dialysis process continued for 1 week until the conductivity of the surrounding water stabilized below 10 μS cm^−1^. The resulting dispersion was acidified with 25 wt% sulfuric acid to protonate all acidic sites, followed by exhaustive washing through centrifugation cycles with methanol to remove all sulfuric acid. Finally, the material was freeze dried and used for characterization and subsequent experiments. Graphenes functionalized with homotaurine (G–HTA) and sulfanilic acid (G–SA) were prepared similarly to the above procedure except for using homotaurine and sulfanilic acid instead of taurine. G‐TA‐COOH was synthesized by treating the G‐TA with 25% nitric acid for 24 h at 60 °C. Pure G‐COOH was synthesized following a previously reported method.^[^ ^19^ ^]^

### 2.3 Catalyst Activity Testing

#### 2.3.1 Esterification

In a typical experiment, the catalyst (10 mg, 7 mass%) and oleic acid (141.2 mg, 0.5 mmol) were combined in a 2 mL screw‐top vial and subjected to sonication for 30 s to ensure uniform dispersion. Under a nitrogen atmosphere, dry methanol (0.4 mL, corresponding to a methanol‐to‐oil molar ratio of 20:1) was added, and the vial was immediately sealed to prevent air exposure. The reaction mixture was sonicated for an additional 30 s and subsequently heated at 60 °C for 4 h. Upon completion, the mixture was allowed to cool to room temperature, and the catalyst was separated by centrifugation at 20 000 rcf for 5 min. The clear supernatant was carefully withdrawn and analyzed directly by gas chromatography (GC) using methyl hexanoate as an internal standard. For nuclear magnetic resonance (NMR) analysis, the supernatant was stored at room temperature for 2 days to allow solvent evaporation.

#### 2.3.2 Recyclability

The catalytic reaction was performed by mixing the G‐TA catalyst (25 mg, 7 mass%) with oleic acid (353 mg, 1.25 mmol) in a 2 mL screw‐top vial, followed by sonication for 30 s to ensure homogeneity. Under an inert nitrogen atmosphere, dry methanol (1.0 mL) was added to the vial, which was then sealed immediately to maintain an oxygen‐free environment. The mixture was sonicated for an additional 30 s and subsequently heated at 60 °C for 4 h. After cooling to room temperature, the catalyst was separated by centrifugation at 20 000 rcf for 5 min. The supernatant was carefully removed and directly analyzed using gas chromatography (GC) with methyl hexanoate as the internal standard. The catalyst was recovered after sequential washing with acetone (2 × 2 mL) and methanol (1 × 2 mL) and then dried overnight in a desiccator under vacuum before reuse in subsequent catalytic cycles.

#### 2.3.3 Transesterification

In a typical experiment, the G‐TA catalyst (20 mg, 7 mass%) and oil (282.4 mg, rapeseed oil, triacetin, tributyrin, or triolein) were combined in a 2 mL screw‐top vial and subjected to sonication for 30 s to ensure homogeneity. Under a nitrogen atmosphere, dry methanol (0.8 mL) was added to the vial, which was immediately sealed to maintain an inert environment. The reaction mixture was sonicated for an additional 30 s and then heated at the desired temperature for the specified duration. Upon completion of the reaction, hexane (1 mL) and water (0.5 mL) were added to the vial, and the mixture was shaken thoroughly to facilitate the separation of glycerol produced during the reaction. The phases were separated by centrifugation at 20 000 rcf for 5 min, and the supernatant (hexane phase) was collected. The collected supernatant was stored at room temperature for 2 days to allow solvent evaporation before being subjected to nuclear magnetic resonance (NMR) analysis.

#### 2.3.4 NMR Yield Calculation

The yield of the esterification reaction was calculated by analyzing the methyl esters in the reaction mixture using quantitative ^1^H NMR.^[^ ^20^, ^21^ ^]^ The methoxy group in the methyl esters at 3.7 ppm (singlet) and the α‐carbonyl methylene groups present in the fatty ester derivatives at 2.3 ppm (triplet) are chosen for integration. CDCl_3_ solutions of known amounts of oil and methyl esters were used for calibration. The transesterification yield (Y) was obtained directly from the area (A) of the selected signals

(1) $$Y\%=100\times \frac{2\times A1}{3\times A2}$$

A1 and A2 are the areas of the methoxy and the methylene protons, respectively.

TOF was determined using the following equation

(2) $$\text{TOF }=\;\frac{\text{Oleic acid converted }\left(\text{mmol}\right)}{\text{catalyst's acidic sites}\left(\text{mmol}\right)\;\times \text{ reaction time }\left(h\right)}\;{\text{in h}}^{\text{−1}}$$

The number of acidic sites was determined from titration results.

Specific productivity was calculated using the equation below, offering unambiguous reaction rates free from potential inaccuracies in active site estimations. This metric is better suited for direct comparisons

(3) $$\text{SP =}\;\frac{\text{Oleic acid converted }\left(\text{mmol}\right)}{\text{total catalyst amount }\left(g\right)\;\times \text{ reaction time }\left(h\right)}\;{\text{in mmol g}}^{\text{−1}}h^{\text{−1}}$$

#### 2.3.5 Carbohydrate Dehydration Reaction

Fructose dehydration: In a typical experiment, 2 mmol fructose, the catalyst (25 mg), and 3 mL dimethyl sulfoxide were mixed in a 10 mL pressure reactor and sonicated for 30 s. Then, under an N_2_ atmosphere, the vial was closed with the screw top and heated at 100 °C for 2 h. After the completion of the reaction, the reaction temperature was brought down to room temperature, and the reaction mixture was separated from the catalyst by centrifugation (20 000 rcf) for 5 min. The product was extracted with ethyl acetate and analyzed directly by GC.

Xylose dehydration: In a typical experiment, the 2 mmol xylose, the catalyst (25 mg), 1.5 mL water, and 1.5 mL tetrahydrofuran were mixed in a 10 mL pressure reactor and sonicated for 30 s. Then, under an N_2_ atmosphere, the vial was closed with the screw top and heated at 130 °C for 5 h. After the completion of the reaction, the reaction temperature was brought down to room temperature, and the reaction mixture was separated from the catalyst by centrifugation (20 000 rcf) for 5 min. The product was extracted with ethyl acetate and analyzed directly by GC.

#### 2.3.6 Computational Details

The density functional theory (DFT) calculations were performed using the CBS‐4M method^[^ ^22^ ^]^ as implemented in the software Gaussian 16.^[^ ^23^ ^]^ The universal continuum solvation model based on solute electron density^[^ ^24^ ^]^ (SMD) was applied to account for solvation effects. The finite‐size model of functionalized ovalene was used (Figure 1) to represent G‐TA, G‐SA, and G‐HTA. The p*K*a values were calculated according to the literature.^[^ ^25^ ^]^

### 2.4 Characterization

High‐resolution transmission electron microscopy (HR‐TEM) and scanning transmission electron microscopy (STEM) in high‐angle annular dark‐field (HAADF) mode for elemental mapping were performed using an FEI TITAN G2 60–300 microscope. The microscope was equipped with an X‐FEG emission gun (300 kV), a spherical aberration corrector for the objective lens, and a ChemiSTEM energy‐dispersive X‐ray spectroscopy (EDS) detector.

X‐ray photoelectron spectroscopy (XPS) was conducted using a PHI VersaProbe II spectrometer (Physical Electronics) with an Al Kα source (15 kV, 50 W). Data analysis was performed using the MultiPak software package (Ulvac‐PHI, Inc.). The fresh G‐TA catalyst was pretreated with the catalytic reaction reagents, thoroughly washed, and subjected to XPS measurements.

X‐ray diffraction (XRD) patterns were recorded with a PANalytical X’Pert PRO MPD (PANalytical, The Netherlands) diffractometer in the Bragg‐Brentano geometry, Co‐Kα radiation (40 kV, 30 mA; λ = 0.1789 nm) equipped with an X’Celerator detector and programmable divergence and diffracted beam antiscatter slits. The measurement range was 2*θ*: 5°–105°, with a step size of 0.033°. The identification of the crystalline phases was performed using the High Score Plus software (PANalytical) which includes the PDF‐4+ database.

Fourier transform infrared (FT‐IR) spectroscopy was carried out using an iS5 FTIR spectrometer (Thermo Nicolet) equipped with a Smart Orbit ZnSe ATR accessory. For the sample preparation, a droplet of an ethanol dispersion of the material was placed on a ZnSe crystal, dried to form a thin film, and analyzed. Spectra were acquired by summing 64 scans under a nitrogen gas flow through the ATR accessory, with ATR and baseline corrections applied.

Raman spectra were recorded using a DXR Raman microscope with a diode laser at an excitation wavelength of 532 nm.

The specific surface area (SSA) and pore size analysis were performed through N_2_ adsorption/desorption measurements at 77 K on a volumetric gas adsorption analyzer (Autosorb iQ XR, Anton‐Paar Quanta Tec, USA) up to 0.965 relative pressure. Before the analysis, the sample was degassed under high vacuum (10 − 7 Pa) at 130 °C for 12 h, while high purity (99.999 %) N_2_ and He gases were used for the measurements. The SSA was evaluated using the Brunauer–Emmett–Teller model based on the Rouquerol criteria^[^ ^26^ ^]^ for N_2_ isotherm. The pore size distribution and pore volume were determined using quenched solid density functional theory (QSDFT).

The wetting behavior was assessed through contact angle analysis of compressed films. Contact angles were measured on a Krüss drop shape analyzer (DSA) controlled with KRÜSS ADVANCE 1.4.1.2 software. The contact angle (*θ*) was calculated for a sessile drop using the Young–Laplace equation 50 μL of OA was automatically dosed on pressed film by Hamilton syringe.

Thermogravimetric analysis (TGA) was performed using a Netzsch STA 449C Jupiter thermal analyzer in synthetic air (100 cm^3^ min^−^^1^). Measurements were conducted in an open α‐Al_2_O_3_ crucible, from 45 to 1000 °C, at a heating rate of 10 °C min^−^^1^.

NMR spectra of esterification products were recorded on a JEOL 400 MHz NMR spectrometer.

The acidic site concentration of the catalyst was determined via acid‐base titration. An aqueous NaOH solution (0.05 M, 10 mL) was added to 80 mg of the catalyst. The mixture was sonicated for 60 min and then stirred at room temperature for 12 h. After centrifugation, 5 mL of the supernatant was titrated with 0.05 M aqueous HCl using phenol red as a pH indicator.

## 3 Results and Discussion

### 3.1 Structural Properties

The G‐TA, G‐SA, and G‐HTA catalysts were prepared starting from GrF, which was sonicated/exfoliated to obtain FG, followed by reaction at 130 °C with the respective nucleophilic aminosulfonic acid molecules (Figure 1a). The nucleophilic amino groups attack the electrophilic centers in FG leading to defluorination and covalent functionalization.^[^ ^18^, ^27^ ^]^ The different solid‐state acid catalysts with homotaurine (HTA, Figure 1b) and sulfanilic acid (SA, Figure 1d) were synthesized to better understand the origin of the activity of the G‐TA catalyst, which previously showed its effectiveness in the acid‐catalyzed reaction of glycerol acetylation.^[^ ^18^ ^]^ In addition, carboxylic acid functionalized graphene (G‐COOH, Figure 1d) and the bifunctional graphene having both –SO_3_H and –COOH groups (G‐TA‐COOH, Figure 1e) were synthesized for comparisons. The catalyst synthesis reaction was monitored with Fourier‐transform infrared (FT‐IR, Figure 1f), indicating the disappearance of CF and CF_2_ bands of the parent FG (at 1200 and 1315 cm^−^^1^, respectively) and the emergence of characteristic aromatic carbon ring bands at 1570 and 1190 cm^−^^1^.^[^ ^28^, ^29^ ^]^ This indicates the almost complete defluorination of FG and the formation of the sp^[^ ^2^ ^]^ network in the products. The 1190 cm^−^^1^ band in the products originates from the aromatic rings^[^ ^29^ ^]^ and not from the C—F bonds, as in the parent FG, also confirmed by the XPS results, discussed later, showing more than 95% fluorine elimination in all products (Table S1, Supporting Information). Furthermore, FT‐IR clearly showed the presence of the SO_3_H group in G‐TA, G‐HTA, G‐SA, and G‐TA‐COOH catalysts, due to its characteristic O = S = O (1190 cm^−^^1^) and SO_3_^−^ (1035 cm^−^^1^) symmetric stretching vibrations observed in FT‐IR.^[^ ^9^, ^30^ ^]^ A broad feature between 3600 and 2900 cm^−^^1^ arises from the presence of N—H and C—H groups and H—O—H molecules. The band at 1725 cm^−^^1^ corresponds to the C=O stretching of the carboxylic groups present in the G‐COOH and G‐TA‐COOH.^[^ ^19^ ^]^ FTIR spectra of TA, HTA, and SA molecules used for the graphene functionalization are shown in Figure S1, Supporting Information, for comparison.

XPS analysis of FG, G‐TA, G‐HTA, and G‐SA confirmed the defluorination and functionalization as evidenced by a decrease in fluorine content from more than 50 at.% in the parent FG^[^ ^31^, ^32^ ^]^ to less than 6 at.% (Figure S2 and Table S1, Supporting Information). The products contained substantial amounts of S, O, and N elements originating from the out‐of‐plane TA, SA, and HTA molecular functionalities. G‐HTA has the highest S content of 4.1%, followed by G‐TA with 3.5% and G‐SA with 2.6%. Interestingly, the S content follows the nucleophilicity of the three molecules. The total acid site density, determined by titration (in mmol g^−1^), suggests higher acidity for the G‐TA derivative (Table S1, Supporting Information). The BET surface area and pore analyses were performed to probe any significant structural differences in the solid state (Table S2, Supporting Information and comments therein).

The Raman spectra (Figure S3, Supporting Information) of the functionalized graphenes displayed the typical vibrations: the G‐band at ≈1580 cm^−^^1^ (representing the vibration of the E2g symmetry mode in graphene) and the D‐band (1350 cm^−^^1^), indicative of structural defects and sp^3^‐hybridized carbon bonds, confirm successful functionalization. The broad character of the D‐band and the high *I*_D_/*I*_G_ ratios (1.06–1.26) suggest high functionalization degrees.^[^ ^33^ ^]^ The XRD pattern of G‐TA, G‐HTA, and G‐SA (Figure S4, Supporting Information) showed a broad 2*θ* reflection at around 28°, suggesting the organization of some areas with a turbostratic arrangement. In parallel, TEM analysis revealed few‐layered transparent flakes with a lateral size of ≈1 μm (Figure S5 and S6a, Supporting Information). Higher‐resolution images of the G‐TA catalyst confirmed the disordered structure due to the high degree of functionalization (Figure S6b). Energy‐dispersive X‐ray analysis (EDS, Figure S6c, Supporting Information) and elemental mapping with high‐angle annular dark‐field scanning transmission electron microscopy (HAADF‐STEM, Figure S6c–h, Supporting Information) also confirmed the presence and homogeneous distribution of the C, N, O, F, and S elements throughout the graphene layers.

The contact angle measurements of G‐TA, G‐HTA, and G‐SA with oleic acid (OA) illustrate variations in surface wettability (Table S2, Supporting Information), with G‐TA exhibiting the lowest value (29.8°), G‐HTA (41.2°), and G‐SA (43.0°), suggesting better OA adsorption for the G–TA derivative. The TGA of G‐TA, G‐HTA, and G‐SA revealed differences in their thermal stability (Figure S7, Supporting Information). Below 200 °C, a minor weight loss is observed for all samples, attributed to the removal of adsorbed moisture and volatile impurities. Between 200 and 400 °C, significant mass loss occurs, with G‐SA exhibiting the highest weight loss, followed by G‐HTA, while G‐TA shows the least loss, indicating superior thermal stability. Beyond 400 °C, gradual decomposition continues, due to the breakdown of residual organic groups and degradation. Together, these results demonstrate that the successful functionalization of graphene with aminosulfonic acid derivatives enhances Brønsted acidity, promotes reactant‐catalyst interactions, and directly correlates with the catalytic performance.

### 3.2 Catalysis

**Figure 2:**
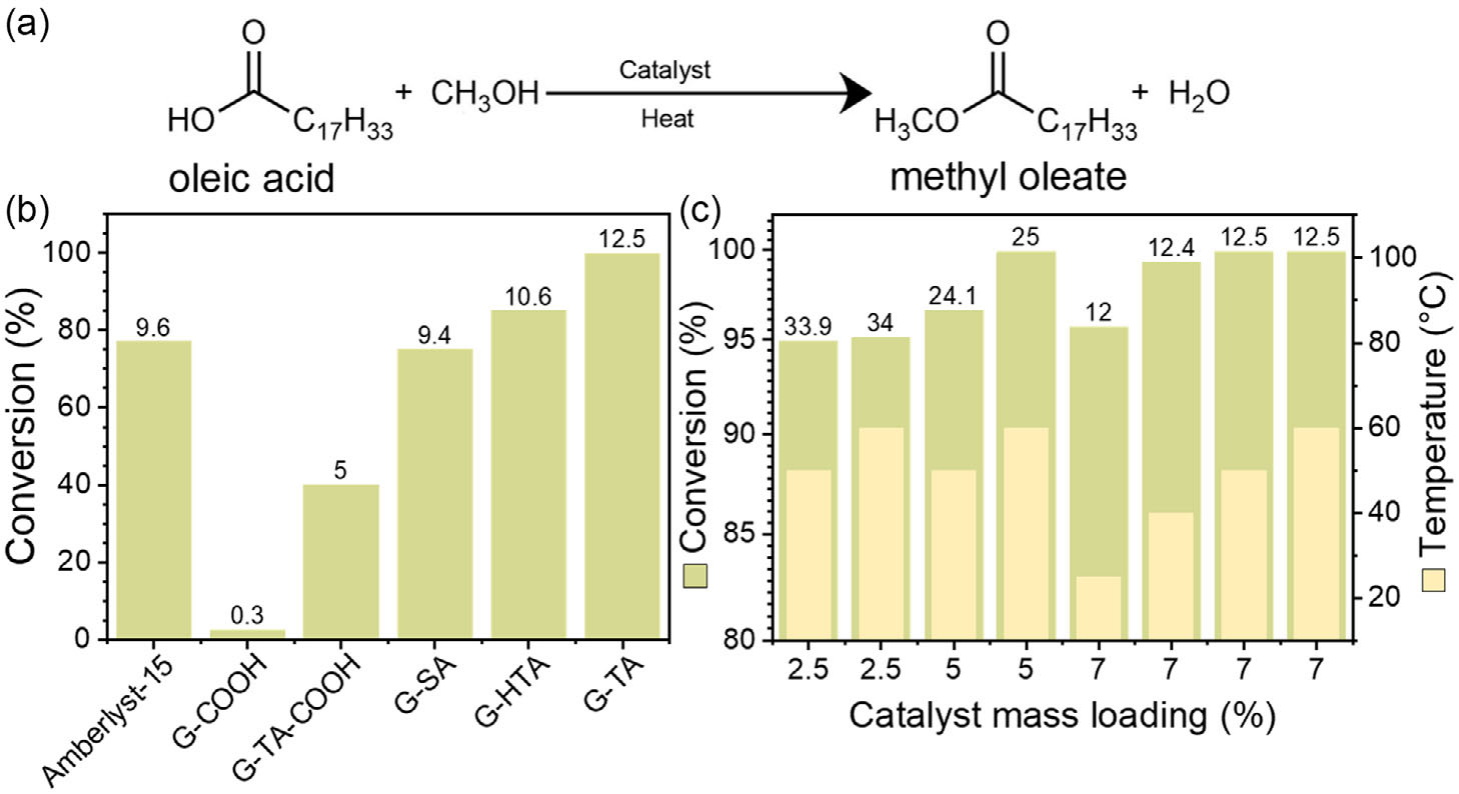
a) Esterification reaction of oleic acid with methanol. b) Esterification of oleic acid with different acid‐functionalized graphenes and with Amberlyst–15. All reactions were performed with a catalyst loading of 7 mass% with respect to oleic acid, at 60 °C. The numbers above the bars represent the SP values (mmol g^−^^1^ h^−^^1^). TOF values are reported in Figure S9, Supporting Information. c) Esterification of oleic acid with different mass% loadings of G‐TA. The numbers above the bars represent SP (mmol g^−^^1^ h^−^^1^). Reaction conditions: oleic acid = 0.5 mmol, methanol =10 mmol, catalyst = 2.5–7 mass% with respect to oleic acid, continuous stirring at 25–60 °C for 4 h.

The catalysts were tested (conversions, selectivities, SP, and TOF were determined) for the esterification reaction of oleic acid with methanol (**Figure** **2**a) at 60 °C and 4 h, along with the benchmark solid‐state sulfonated catalyst of Amberlyst (Figure 2b and Figure S8, Supporting Information). The aminosulfonated catalysts (G‐SA, G‐HTA, and G‐TA) displayed the highest yields and SP particularly in terms of activity according to the active sites (TOF, Figure S9, Supporting Information). Amberlyst displayed similar SP; however, its TOF was half in comparison to the aminosulfonated graphene derivatives, due to its higher content in acidic sites. The substantially higher activity of all the aminosulfonated catalysts verifies the initial hypothesis that the combination of amino and sulfonic acid groups leads to particularly high acidity and, thus, activity. The high acidity of all the aminosulfonated catalysts was theoretically verified as the p*K*a values were −7.2, −6.3, and −6.5, for G‐SA, G–HTA, and G‐TA, respectively, stronger by 8 p*K*a units in comparison to the same derivative but without the amino group.^[^ ^18^ ^]^ The activity of such solid‐state catalysts may also be affected by the presence of other less acidic oxygen groups^[^ ^14^ ^]^ or by the presence of metal impurities. However, the consistently very high TOF of all the amino/sulfonated derivatives verifies the origin of their activity and excludes any significant role of other chemical groups (e.g., oxygen from the environment or residual fluorine). To further exclude the role of random metal impurities or other chemical groups, the graphene acid catalyst with selective carboxylic functionalities was also tested and synthesized under the identical laboratory environment and procedures,^[^ ^19^ ^]^ showing negligible activity (Figure 2b) in line with the lower acidity of the carboxylic groups. However, upon the addition of taurine in the dual‐functionalized catalyst (G‐TA‐COOH) the yield sharply increased to 40% (Figure 2b), verifying the key role of the functional groups rather than other random impurities. All the aminosulfonic acid derivatives exhibited high activities, reaching 75% conversion for G‐SA (with TOF = 3.9 h^−^^1^), 85% for G‐HTA (TOF = 3.8 h^−^^1^), and 99.9% conversion for G‐TA (TOF = 4.1 h^−^^1^), at 60 °C for 4 h. The G‐TA showed complete conversion (99.9%) also at 50 °C, while keeping 99.3% conversion even at 40 °C (Figure 2c), substantially outperforming the benchmark catalyst of Amberlyst‐15 (with 8.6 at. % content of sulfur, TOF = 2 h^−^^1^ and 77% conversion at 60 °C). G–TA was selected for further studies because of its slightly higher TOF and SP but also due to the improved interaction with oleic acid and thermal stability in comparison to the G‐SA and G‐HTA.

**Figure 3:**
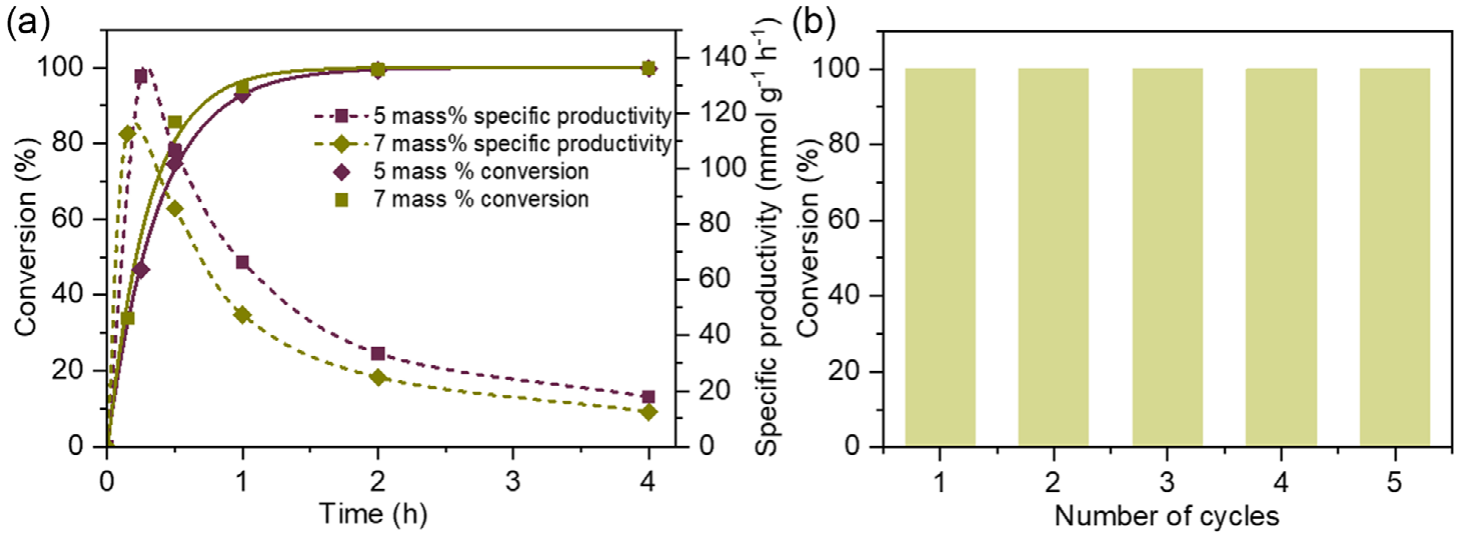
a) Kinetics of the oleic acid esterification with methanol using the G‐TA catalyst at loadings of 5 and 7 mass%. b) Recycling performance of the 7 mass% G‐TA catalyst for the oleic acid esterification. Reaction conditions: oleic acid = 0.5 mmol, methanol = 10 mmol, with continuous stirring at 60 °C for 4 h.

The G‐TA catalyst was thus further studied under different reaction conditions with varying catalyst mass loadings and reaction temperatures (Figure 2c). A catalyst loading of 2.5 mass% with respect to the oleic acid converts 94.9% of oleic acid at 50 °C with an SP of 33.9 mmol g^−^^1^ h^−^^1^. A further increase in the catalyst loading to 7 mass% shows 95.7% oleic acid conversion even at room temperature (25 °C), and conversion increased to above 99% at 40 °C. Based on the kinetics (**Figure** **3**a), the highest TOF and SP at the first steps of the reaction were 52.2 h^−^^1^ and 133.4 mmol g^−^^1^ h^−^^1^, respectively. The recyclability studies (Figure 3b) indicated that the G‐TA catalyst remained remarkably stable in the esterification reaction, with no notable changes in conversion over five consecutive reaction cycles, as confirmed by XPS analysis of the recovered catalyst after five cycles (Figure S10, Supporting Information).

**Figure 4:**
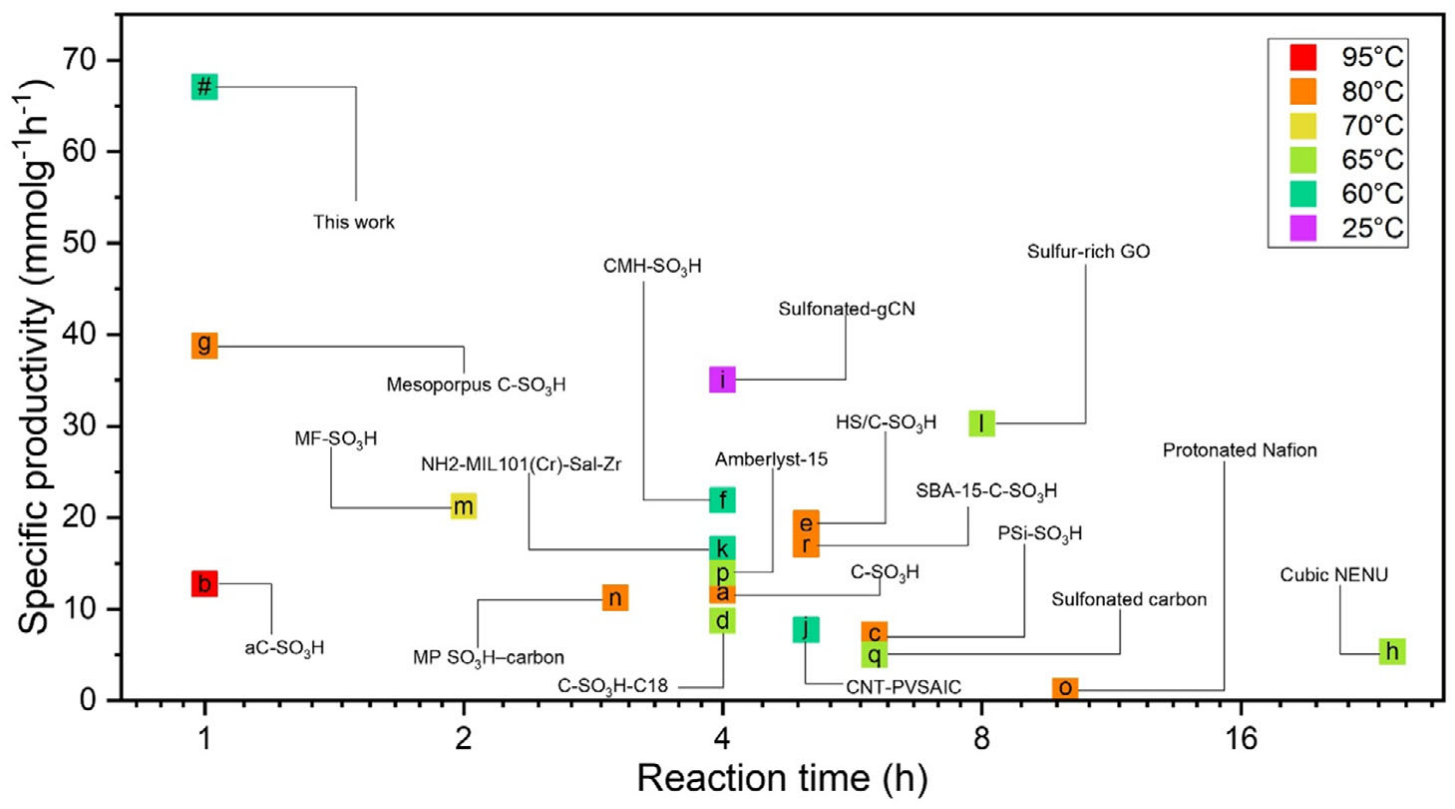
Specific productivities of the G‐TA catalyst for 1 h and 4 h of reaction for oleic acid esterification, and the respective specific productivities of previously reported heterogeneous catalysts. The colors represent the temperature of the reaction. a,^[^ ^13^ ^]^ b,^[^ ^9^ ^]^ c,^[^ ^15^ ^]^ d,^[^ ^66^ ^]^ e,^[^ ^67^ ^]^ f,^[^ ^14^ ^]^ g,^[^ ^35^ ^]^ h,^[^ ^67^ ^]^ i,^[^ ^68^ ^]^ j,^[^ ^69^ ^]^ k,^[^ ^70^ ^]^ l,^[^ ^71^ ^]^ m,^[^ ^72^ ^]^ n,^[^ ^73^ ^]^ o,^[^ ^73^ ^]^ p,^[^ ^74^ ^]^ q,^[^ ^34^ ^]^ r.^[^ ^67^ ^]^

To interpret and evaluate the present results within the broader context of the state‐of‐the‐art, extensive data were collected on heterogeneous acid catalysts for oleic acid esterification with methanol (Table S3, Supporting Information, and **Figure** **4**). For a clear comparison regarding the practicality of the catalyst, we considered SP values, which rely on the total mass of the catalyst used in the reaction, but TOF values are also provided for a clearer picture. SP is crucial for evaluating overall catalyst efficiency, particularly in reactions where the active centers are not well known, while TOF is better for understanding the activity of the actual active sites. Toda et al. achieved a 100% yield and an SP of 12.5 mmol g^−^^1^ h^−^^1^ (“a” in Figure 4 and Table S3, Supporting Information) in the esterification of oleic acid and stearic acid at 80 °C using sulfonated amorphous carbon derived from sugars.^[^ ^13^ ^]^ Carbon‐based mesoporous solid acid catalysts achieved an SP of 21.9 mmol g^−^^1^ h^−^^1^ at 60 °C, (“f” in Figure 4 and Table S3, Supporting Information).^[^ ^14^ ^]^ Polystyrene sulfonated resins and carbons achieved less than 13 mmol g^−^^1^ h^−^^1^.^[^ ^9^, ^15^, ^34^ ^]^ Microporous carbons with SO_3_H groups have demonstrated outstanding catalytic activity with SP of 38.8 mmol g^−^^1^ h^−^^1^ (“g” in Figure 4 and Table S3, Supporting Information) at 80 °C.^[^ ^34^, ^35^ ^]^ However, the current G‐TA catalyst efficiently converts oleic acid at 60 °C with particularly high efficiency, reaching 67 mmol g^−^^1^ h^−^^1^ at 1 h with 94% yield or SP = 47 mmol g^−^^1^ h^−^^1^ at 1.5 h with 99% yield (Figure 4), and exhibited one of the highest TOF values (Table S3, Supporting Information).

According to the reaction mechanism (Figure S11, Supporting Information), the acid catalysts involve the protonation of oxygen on the carbonyl carbon by the Brønsted acid sites of the catalyst. DFT calculations further corroborated that the synergistic interplay of amino and sulfonic groups in these catalysts generated exceptional Brønsted acidity, consistent with their observed reactivity trends. Certainly, the carboxyl‐based catalyst G–COOH exhibits substantially lower acidity strength (p*K*a=5.2);^[^ ^19^ ^]^ thus, it is almost inactive (Figure 2b). Partial functionalization of the G‐GOOH with taurine molecules sharply increases the activity (G–TA–COOH; Figure 2b). These control studies highlight the importance of rational catalyst design, and the advantages offered in terms of acidity using such aminosulfonic acid derivatives. This is further supported by DFT calculations that were performed for the G‐TA catalyst;^[^ ^18^ ^]^ when the amino group of taurine was replaced with a methylene group, the acidity dramatically reduced, unveiling the key role of the combined aminosulfonate group presence on the G‐TA catalyst. In particular, the presence of the ammonium group was responsible for a profound increase in the acidity of G‐TA with a p*K*a lower by ≈8 units in comparison to the methylene_‐_substituted analog. The high acidity greatly facilitates the proton exchange between the substrate and reagents/intermediates enhancing the overall catalytic performance.

**Figure 5:**
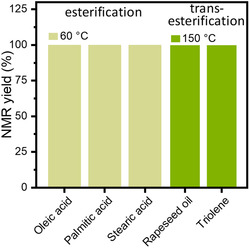
NMR yields of various fatty acids and vegetable oils esterification and transesterification reactions. Reaction conditions: 280 mg oil/fatty acid, 20 mmol of methanol, 7 mass% catalyst, with continuous stirring at 60 or 150 °C for 4 h.

To further evaluate the potency and broad application potential of the G‐TA catalyst its activity was also tested for the transesterification of vegetable oils, which is an essential industrial reaction in manufacturing plasticizers, biofuels, and for plastic recycling.^[^ ^35^, ^36^, ^37^, ^38^ ^]^ G–TA converted more than 99% of triacetin within 4 h (Figure S12a, Supporting Information). In the case of tributyrin transesterification, 96% conversion was achieved after around 12 h (Figure S12b, Supporting Information). The longer time required in comparison to esterification is due to the increased fatty acid chain length causing steric hindrance^[^ ^39^ ^]^ and due to the slower acid‐catalyzed alcoholysis.^[^ ^40^, ^41^ ^]^ However, this can be improved by using higher temperature, pressure, and higher methanol to triglyceride ratio.^[^ ^35^, ^42^, ^43^, ^44^, ^45^ ^]^ Thus, the yield of methyl esters from rapeseed oil and triolein reached over 99.9% at 150 °C (**Figure** **5** and Figure S13, Supporting Information).

Hara et al. reported highly active sulfonated carbon catalysts^[^ ^42^, ^46^ ^]^ which produced ≈8% yield of methyl oleate from triolein at 130 °C in 4 h. G‐TA produced the same 8% yield at 100 °C within 4 h, demonstrating the potency of the present catalyst. A comparable catalyst, derived via sulfonating carbonized glucose (but in fuming H_2_SO_4_), showed ≈24% yield at 150 °C (SP = 3.6 mmol g^−^^1^ h^−^^1^, 3 mmol triolein, 0.2 g catalyst, 3 h).^[^ ^37^, ^47^ ^]^ In another work, Bitter et al. used aryl sulfonic acid‐grafted carbon nanofibers catalysts for triolein transesterification,^[^ ^45^ ^]^ yielding 73% product in 4 h at 120 °C, reaching an SP of 12 mmol g^−^^1^ h^−^^1^, similar as the G‐TA catalyst, but at 150 °C. Importantly, G‐TA was similarly effective in transforming crude rapeseed oil (Figure 5), which, unlike triolein, contains a broad variety of triglycerides and other substances.

To further highlight the acidity‐induced high catalytic activity of G‐TA, the broader applicability and scope were demonstrated for the conversion of biomass‐derived carbohydrates to value‐added chemicals, such as furfural and hydroxymethylfurfural.^[^ ^48^, ^49^, ^50^ ^]^ Therefore, valorization of waste biomass‐derived carbohydrates substantially contributes to meeting the sustainability goals in chemical synthesis and fuel production.^[^ ^51^ ^]^ Acid‐functionalized materials, such as sulfonated hydrochars,^[^ ^52^, ^53^, ^54^ ^]^ sulfonated graphene,^[^ ^55^, ^56^ ^]^ nanotubes,^[^ ^57^ ^]^ mesoporous carbons,^[^ ^57^, ^58^, ^59^ ^]^ and carbon nitrides^[^ ^60^ ^]^ (Table S4, Supporting Information), are effective catalysts for dehydrating C5–C6 sugars.^[^ ^53^, ^55^, ^57^, ^58^, ^60^, ^61^ ^]^ A preliminary study, without optimizing reaction conditions (100 °C), showed that G‐TA could effectively convert fructose and xylose under mild conditions with competitive performance (Table S4, Supporting Information) compared to existing catalysts for carbohydrate dehydration reactions,^[^ ^62^, ^63^, ^64^, ^65^ ^]^ particularly considering the mild conditions. The G‐TA converted fructose to hydroxymethylfurfural within 2 h, with SP of 40 mmol g^−^^1^ h^−^^1^. Additionally, xylose was converted to furfural in 5 h at 130 °C, achieving over 99% selectivity and yield, with a SP of 16 mmol g^−^^1^ h^−^^1^ (Table S4 and Figure S14, Supporting Information). To address the stability, we performed multiple cycles of carbohydrate dehydration reactions (Figure S15, Supporting Information). The G‐TA catalyst demonstrated good recyclability, maintaining more than 75% yield even after the fifth cycle. We also investigated sulfur leaching using XPS analysis, which revealed that ≈86% of the sulfur content was retained after the fifth cycle at 100 °C (Figure S16, Supporting Information). These findings indicate that the catalyst retains a significant portion of its acidic sulfonic acid functionalities, and even after repeated use exhibits a twofold higher activity than that of the fresh, benchmark Amberlyst catalyst (Figure S15, Supporting Information). The same conclusions are drawn from the TOF values: Amberlyst‐15 showed a TOF of 3.2 h^−^^1^ and G‐TA a TOF of 13.0 h^−^^1^, demonstrating the very good performance of the aminosulfonated catalyst. The results on the transformation of hexoses highlight the broad applicability of the superacidic heterogeneous G‐TA catalyst synthesized without harsh sulfonation, using only the natural amino acid of taurine.

## 4 Conclusion

This work reports aminosulfonated catalysts for the esterification of oleic acid, demonstrating high activity, selectivity, and recyclability for biodiesel synthesis. The key finding lies in the high activity of such derivatives when both the amino and the sulfonate groups are present. These catalysts are synthesized through a benign covalent functionalization of FG, avoiding harsh sulfonation methods (e.g., concentrated H_2_SO_4_ or fuming SO_3_). The study further emphasized the taurine‐functionalized catalyst (2‐aminoethane sulfonic acid), which showed modest improvements over the other aminosulfonated derivatives. Experimental and theoretical studies unveiled the key role of the out‐of‐plane chemical functionalities bearing an amino and sulfonate group, ascribing superior acidity and boosting the catalytic activity. The catalyst exhibited excellent activity and selectivity for converting oleic acid to its methyl ester, offering enhanced performance over benchmark catalysts. The versatility of G‐TA was further demonstrated in other acid‐catalyzed reactions, including the transesterification of vegetable oils and dehydration of carbohydrates. In both cases, G‐TA delivered competitive yields and rates compared to standard and advanced catalysts. The development of such potent, recyclable superacid catalysts is essential, as acid catalysis remains one of the most broadly applicable strategies in organic synthesis, spanning biological and industrial transformations.

## Conflict of Interest

The authors declare no conflict of interest.

## Author Contributions

**Aby Cheruvathoor Poulose**: data curation (lead); formal analysis (lead); investigation (lead); methodology (lead); validation (lead); visualization (lead); writing—original draft (lead). **Hugo Bares**: investigation (supporting); methodology (supporting). **Dagmar Zaoralová**: formal analysis (supporting); investigation (supporting); methodology (supporting); validation (supporting); visualization (supporting). **Ivan Dědek**: data curation (supporting); investigation (supporting); methodology (supporting). **Michal Otyepka**: formal analysis (supporting); funding acquisition (lead); investigation (supporting); methodology (supporting); supervision (equal). **Aristides Bakandritsos**: formal analysis (supporting); funding acquisition (supporting); methodology (supporting); supervision (lead); visualization (equal); writing—original draft (equal); writing—review and editing (lead). **Radek Zbořil**: funding acquisition (lead); supervision (equal); writing—review editing (equal).

## Supporting information

Supplementary Material

## Data Availability

The data that support the findings of this study are openly available in Zenodo at https://doi.org/10.5281/zenodo.15173286.
