# Supplementary material for: Aminosulfonated Graphene as a Catalyst for Efficient Production of Biodiesel from Fatty Acids and Crude Vegetable Oils
Source: ChemSusChem. 2025 Apr 18;18(12):e202402488. doi: 10.1002/cssc.202402488 (PMC12175036; doi:10.1002/cssc.202402488)
Supplement: Supplementary file 1 — Supplementary Material [file CSSC-18-e202402488-s001.pdf]

Supporting Information

Amino-Sulfonated Graphene as a Catalyst for Efficient Production of Biodiesel From Fatty Acids and Crude Vegetable Oils.

Aby Cheruvathoor Poulose,[1,*] Hugo Bares,[1,2] Dagmar Zaoralová,[3] Ivan Dědek,[1,3] Michal Otyepka,[1,3] Aristides Bakandritsos,[1,4,*] Radek Zbořil[1,4*]

[1]Regional Centre of Advanced Technologies and Materials, Czech Advanced Technology and Research Institute (CATRIN), Palacký University in Olomouc, Šlechtitelů 27, 783 71, Olomouc, Czech Republic;

[2]Present address: Lepty, 14 avenue Pey-Berland, 33600, Pessac, France,

[3]IT4Innovations, VŠB−Technical University of Ostrava, 17. listopadu 2172/15, Ostrava, Poruba 708 00, Czech Republic

[4]Nanotechnology Centre, Centre for Energy and Environmental Technologies, VŠB–Technical University of Ostrava, 17. listopadu 2172/15, Poruba, 708 00, Ostrava, Czech Republic

Table S1. Elemental content of the pristine graphite (GrF), graphene functionalized with taurine (G-TA), homotaurine (G-HTA), and sulfanilic acid (G-SA) along with corresponding acid density values from titration.

Elements	GrF	G-TA	G-HTA	G-SA
Carbon	41.6	69.9	66.5	68.8
Nitrogen	0.5	8.9	8.3	7.7
Oxygen	-	17.1	19.6	15.1
Fluorine	57.9	0.6	1.3	5.5
Sulfur	-	3.5	4.1	2.7
Acid density from titration (mmol/g)		3.1	2.7	2.4
Acidity (pK_a)#		-6.5	-6.3	-7.2

#From theoretical calculations

Table S2. Textural Properties and contact angle of G-TA, G-SA, and G-HTA materials.

	G-TA	G-SA	G-HTA
Surface Area (BET)	18.3 $m^2 g^{-1}$	268.1 $m^2 g^{-1}$	14.6 $m^2 g^{-1}$
Pore volume	0.020 $cm^3 g^{-1}$	0.158 $cm^3 g^{-1}$	0.033 $cm^3 g^{-1}$
Average pore size	5.1 nm	12.6 nm	6.8 nm
Contact angle with oleic acid	29.8°	43.0°	41.2°

Comment: The textural properties analysis revealed that G-SA exhibited superior surface area (268.6 m^2/g) and pore volume (0.158 cm^3/g) compared to G-TA and G-HTA samples. The pore sizes ranged from 5.1 to 12.6 nm across the samples, with G-SA showing the largest pore diameter. However, it should be noted that these features obtained through nitrogen adsorption do not fully correspond to the phenomena taking place in the reaction mixture, as these materials are layered systems with sheets that are independent of each other. Thus, in the solvent, the arrangement of the sheets changes, since they may solvate and exfoliate up to a certain extent. Here, we observe that the derivative with the most rigid functionalization (the aromatic molecule of sulfanilic acid) provides a higher surface area and pore volume because of its rigidity, which retains some space between the layers even in the solid state. The aliphatic molecules (taurine and homotaurine) are very flexible, not able to retain the sheets separated, thus the surface areas of the dried solids are very small, in line with many such graphene derivatives, like the nitrile and carboxyl-modified graphenes.[1]

Table S3. Comparison with oleic acid esterification reaction using previously reported heterogeneous catalysts.

		Catalyst	Mass %	Catalyst (g)	Acid density (mmol/g)	Oleic Acid (mmol)	Alcohol: Oil	T (°C)	t (h)	Yield (%)	TOF* (h^{-1})	Space-time yield[#] (h^{-1})	Specific productivity[$] (mmol g^{-1} h^{-1})	Reference
1	a	C-SO_3H	7	0.2	2.5	10	10:1	80	4	100	5.0	3.6	12.5	[2]Nature 2005, 438, 178
2	b	aC-SO_3H	17	0.307	1.8	6.3	25:1	95	1	62	7.1	3.6	12.7	[3]ACS Catal. 2012, 2, 1296
3	c	PSi-SO_3H	12.5	0.05	2.4	2	5:1	80	6	89.7	2.5	1.2	6.0	[4]Nat. Commun. 2014, 5, 3170
4	d	C-SO_3H-C18	24.5	0.25	2.3	10	5:1	65	4	87.3	3.8	0.9	8.7	[5]Journal of Material Chemistry A 2014, 2, 11195
5	e	HS/C-SO_3H	3.5	0.1	2.1	10	10:1	80	5	96.9	9.2	5.5	19.4	[6]ACS Applied Mater. & Interf. 2015, 7, 26767
6	f	CMH-SO_3H	2.8	0.048	1.1	6	3:1	60	4	70	19.9	6.3	21.9	[7]ACS Catal. 2015, 5, 4951
7	g	Mesoporous C-SO_3H	8.5	0.2	0.51	8.25	3:1	80	1	94	76.0	11.1	38.8	[8]ChemCatChem 2015, 7, 3945
8	h	Cubic NENU	2.5	0.035	NA	5	50:1	65	24	90	-	1.5	5.4	[9]J. Am. Chem. Soc., 2015, 137, 12697
9	i	Sulfonated-gCN	2.5	0.025	1.21	3.54	35:1	25	4	99	29.0	9.9	35.0	[10]Scie. Rep. 2016, 6, 39387
10	j	CNT-PVSAIC	7	0.02	1.4	1	30:1	60	5	77.2	5.5	2.2	7.7	[11]ACS Sust. Chem. & Eng. 2016, 4, 3140
11	k	NH_2-MIL101(Cr)-Sal-Zr	4	0.0112	3.48	1	10:1	60	4	74.1	4.8	4.6	16.5	[12]Appl. Surf. Sci., 2017, 412, 394
12	l	Sulfur-rich GO	5	0.27	2.6	71	22:1	65	8	92	11.6	2.3	30.2	[13]ChemSusChem 2017, 10, 3352
13	m	MF-SO_3H	8	0.0226	3.34	1	8:1	70	2	95.9	6.4	6.0	21.2	[14]Fuel 2020, 266, 117149.
14	n	MP SO_3H–carbon	6	0.43	2.3	25	10:1	80	3	58	4.9	3.2	11.2	[15]Catal. Today 2010, 150(1-2) (1-2), 140-146
15	o	Protonated Nafion	17	1.25	0.8	25	10:1	80	10	51	1.3	0.3	1.0	[15]Catal. Today 2010, 150(1-2) (1-2), 140-146
16	p	Amberlyst-15	2	0.05	5.31	3.54	55:1	65	4	79	2.6	9.9	14.0	[16]Catal. Commun. 2011, 13(1) (1), 26-30
17	q	Sulfonated carbon	10	3	0.59	106	20:1	65	6	90	9.0	1.5	5.3	[17]Renew. Energ. 2019, 130, 510-523
18	r	SBA-15-C-SO_3H	3.6	0.1	2.23	10	10:1	80	5	85.2	7.6	4.7	17.0	[6]ACS Applied Mater. & Interf., 7, 26767–26775, 2015
**19**	**#**	**G-TA**	**5**	**0.007**	**3.07**	**0.5**	**20:2**	**60**	**1**	**94**	**21.9**	**18.8**	**67.1**	**This work**

*Turnover frequency (TOF): the moles of product formed over quantity (mmol) of the active site in the catalyst over the time of the reaction

[#]Space-time yield: the amount (mass) of product formed over the total mass of the catalyst utilized in the reaction over the time of the reaction.

[$]Specific productivity: the moles of product formed over the total mass of the catalyst utilized in the reaction over the time of the reaction

Table S4. Comparisons of the G-TA catalyst and the previously reported heterogeneous catalysts for the carbohydrate dehydration reactions.

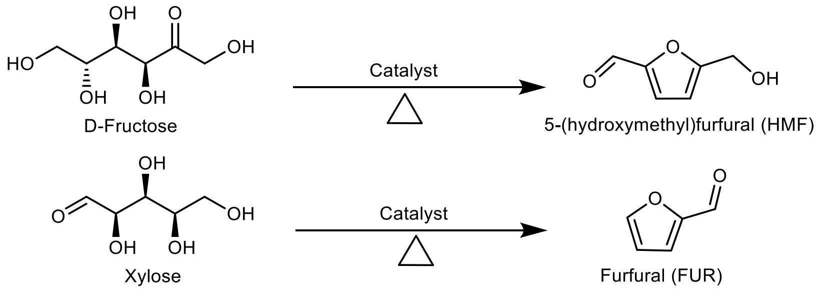

Catalyst	Temperature	Time	Substrate	Solvent	Catalyst concentration	Main reaction product* (yield %)	Specific productivity$ (mmol g^{-1} h^{-1})	Reference.
Starbon®450-SO_3H	200 °C	1 h	Xylose (0.14mmol)	Water/cyclopenth ylmethyl ether	21 mg	FUR (69.5)	4.6	[18]*Applied Catal. A: Gen., 585, 2019, 117180*
Sulfonated sporopollenin	190 °C	40 min	Xylose (1mmol)	Water/cyclopenth ylmethyl ether/NaCl	15 mg	FUR (69.0)	69	[19]*ACS Sust. Chem. Eng. 2017, 5, 1, 392–398*
Sulfonated carbo Mxg	190 °C	1 h	Xylose (1 mmol)	Water/cyclopenth ylmethyl ether	15 mg	FUR (60)	40	[20]Mol. Catal. 2017, 438, 167– 172
γ-Al_2O_3/$CaCl_2$	150 °C	50 min	Xylose (1mmol)	Water-Toluene	50 mg	FUR (55.0)	13	[21]*Applied Catal. A: Gen. 585,2019, 117188*
Zn doped CuO NP	150 °C	12 h	Xylose (1mmol)	Water	7.5 mg	FUR (86.0)	9.5	[22]*Ultrason. Sonochem. 56,2019,55-62*
PFCN	150 °C	5 h	Xylose (0.2 mmol)	Water/THF	50 mg	FUR (91.0)	0.7	[23]ChemSusChem 2020,13, 5231– 5238
LS-SO_3H	140°C	3 h	Fructose (6 mmol)	DMSO	200 mg	HMF (83.1)	8.3	[24]RSC Adv. 2018, 8, 15762– 15772
PhSO_3H-Mesoporous Carbon	140°C	30 min	Fructose (4.16 mmol)	Isopropanol	90 mg	HMF (91)	84	[25]ACS Appl. Mater. Interfaces 2015, 7, 34, 19050–19059
**G-TA**	**130 °C**	**5 h**	**Xylose (2 mmol)**	**Water/THF**	**25 mg**	**FUR (99.0)**	**16**	**This work**
Polystyrene-SO_3H-Carbon	120°C	30 min	Fructose (0.83 mmol)	DMSO	15 mg	HMF (89)	99	[26]Green Chem. 2013, 15, 2895– 2903
Sg-CN	100 °C	30 min	Xylose (2 mmol)	Water	50 mg	FUR (95.0)	152	[27]Green Chem., 2017,19, 164-168
Sulfonated Cellulose derived carbon	80 °C	20 min	Fructose (0.55 mmol)	Butyl methyl imadzolium chloride	50 mg	HMF (76)	25	[28]ChemSusChem 2012, 5, 2215– 2220
**G-TA**	**100 °C**	**24 h**	**Xylose (2 mmol)**	**Water/THF**	**25 mg**	**FUR (99.0)**	**3.3**	**This work**
**G-TA**	**100 °C**	**2 h**	**Fructose (2 mmol)**	**DMSO**	**25 mg**	**HMF (99.0)**	**40**	**This work**

*FUR-Furfural, HMF- hydroxymethylfurfural

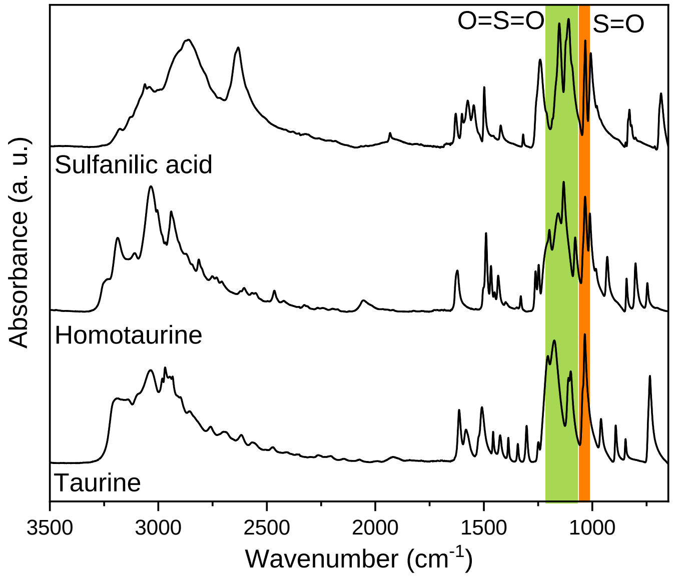

Figure S1. FTIR spectrum of pure taurine, homotaurine and sulfanilic acid.

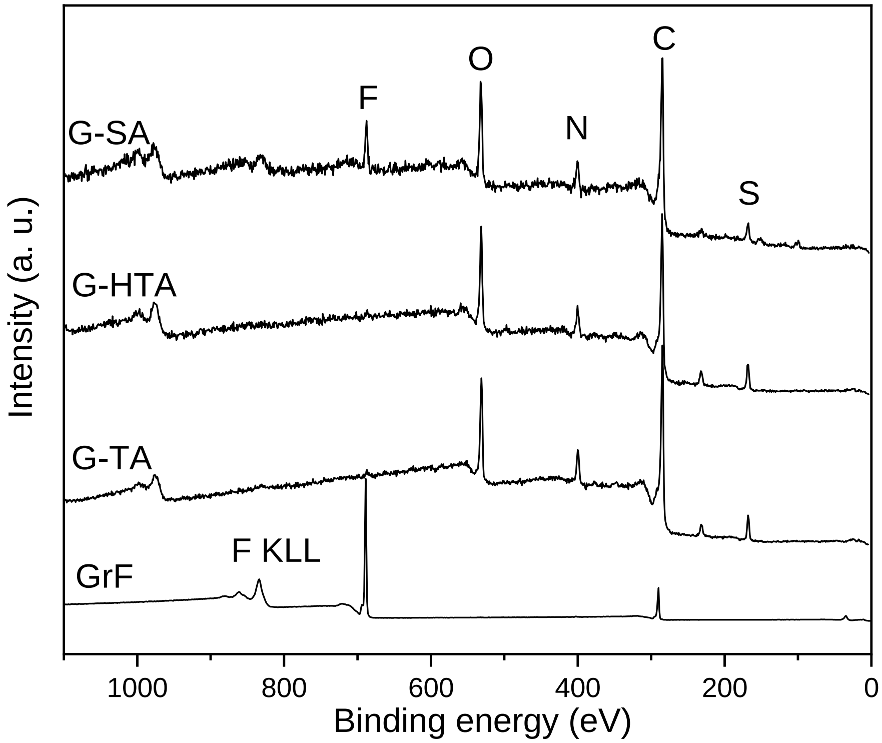

Figure S2. XPS spectra of the pristine graphene (GrF), graphene functionalized with taurine (G-TA), homotaurine (G-HTA), sulfanilic acid (G-SA) and fluorographite (GrF).

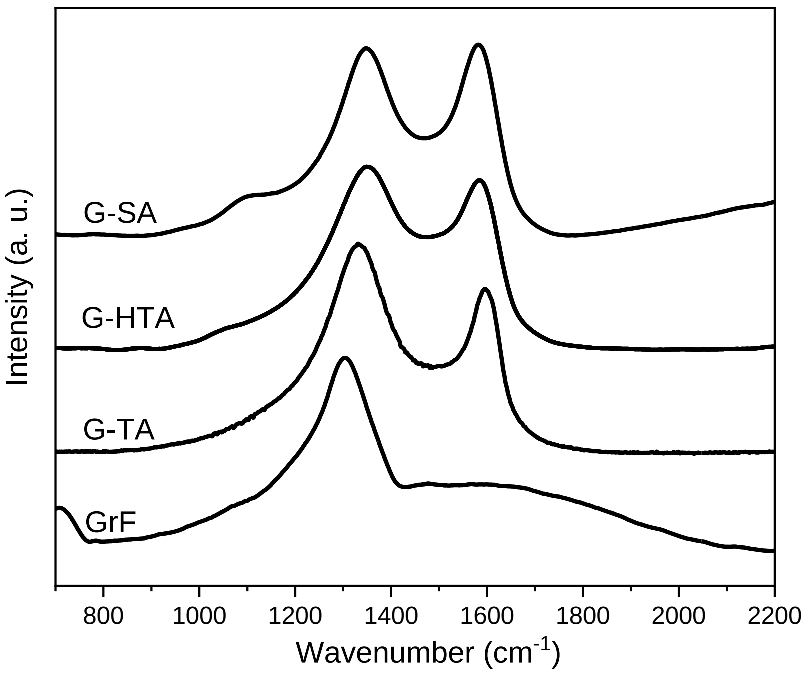

Figure S3. Raman spectra of graphene functionalized with taurine (G-TA), homotaurine (G-HTA), sulfanilic acid (G-SA), and fluorographite (GrF).

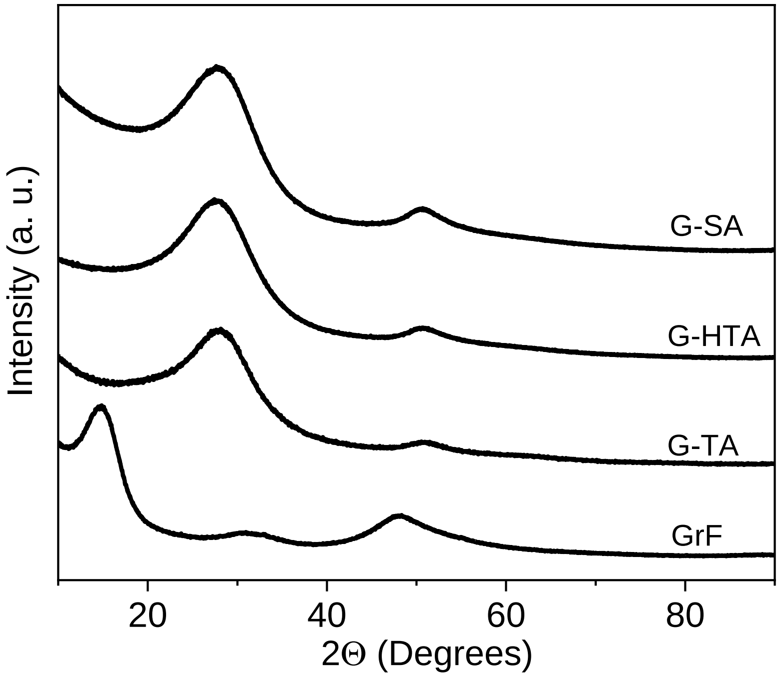

Figure S4. XRD of graphene functionalized with taurine (G-TA), homotaurine (G-HTA), sulfanilic acid (G-SA), and fluorographite (GrF).

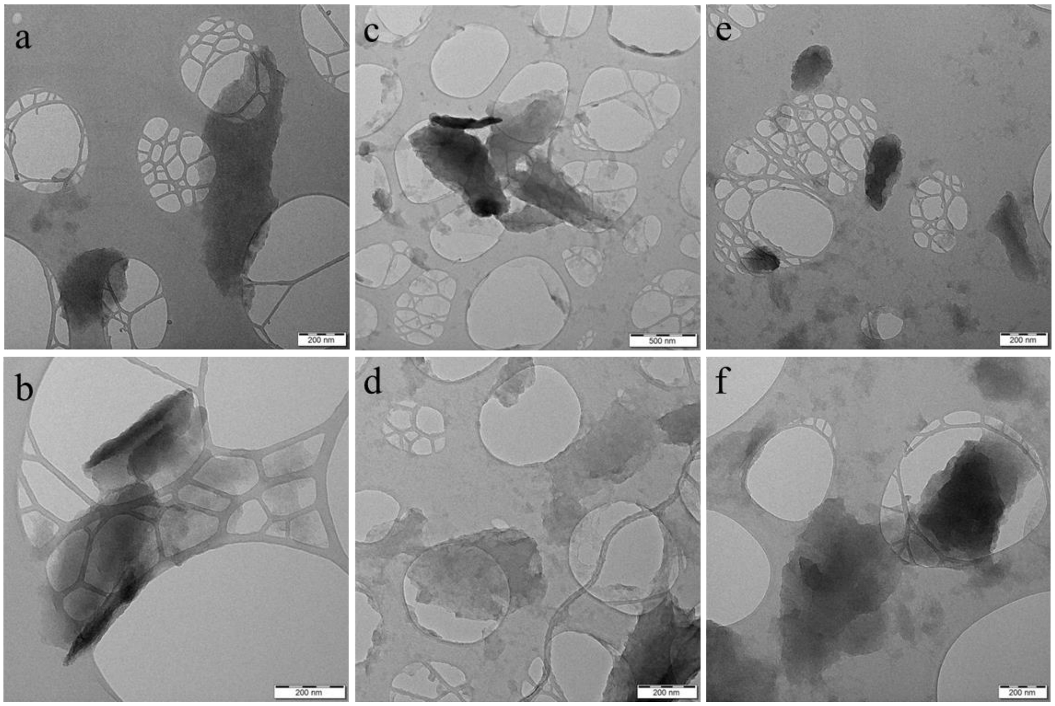

Figure S5. TEM images of graphene functionalized with (a-b) taurine, (c-d) homotauine, and (e-f) sulfanilic acid.

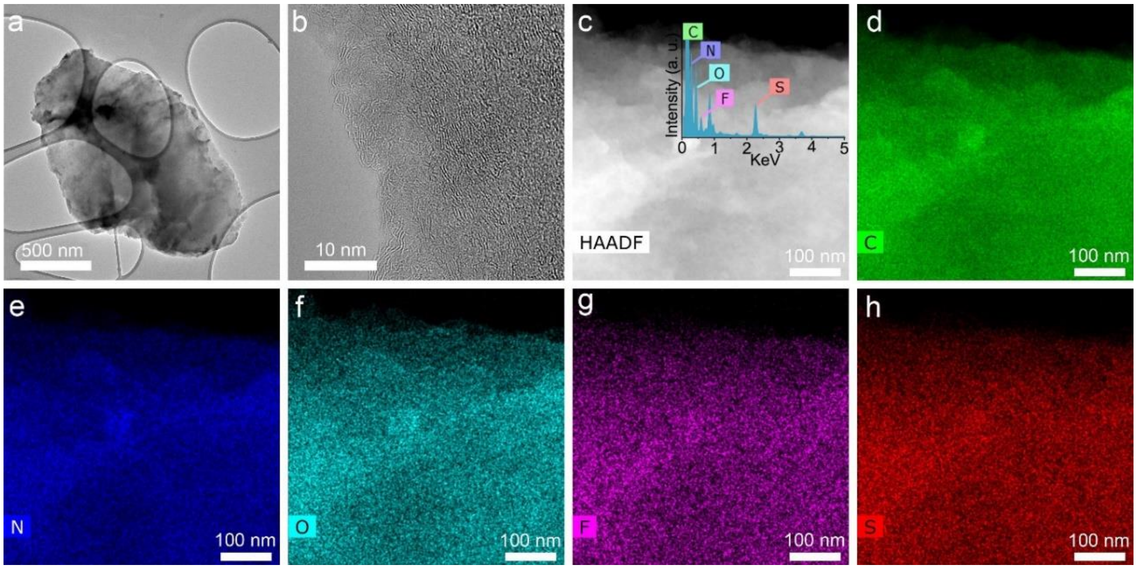

Figure S6. (a, b) High-resolution transmission electron micrographs of few-layered flakes of the G-TA catalyst. (c) High-angle annular dark-field scanning transmission electron micrographs of the flake and the corresponding energy dispersive X-ray analysis spectrum (inset), along with X-ray chemical mapping for (d) carbon, (e) nitrogen, (f) oxygen, (g) fluorine, and (h) sulfur.

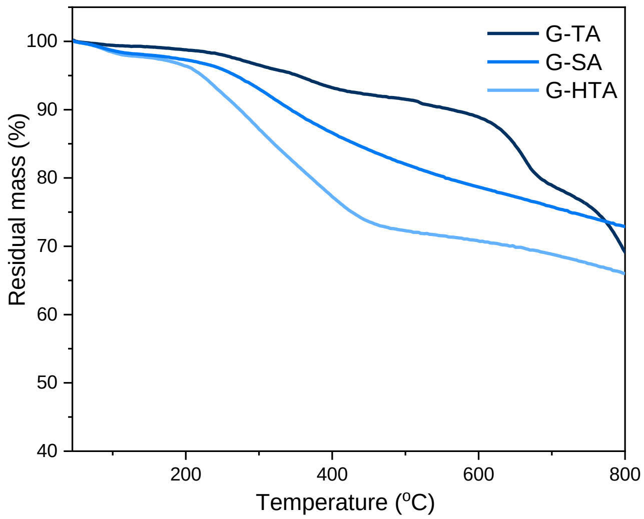

Figure S7. Thermal stability of G-TA, G-HTA, and G-SA materials according to TGA.

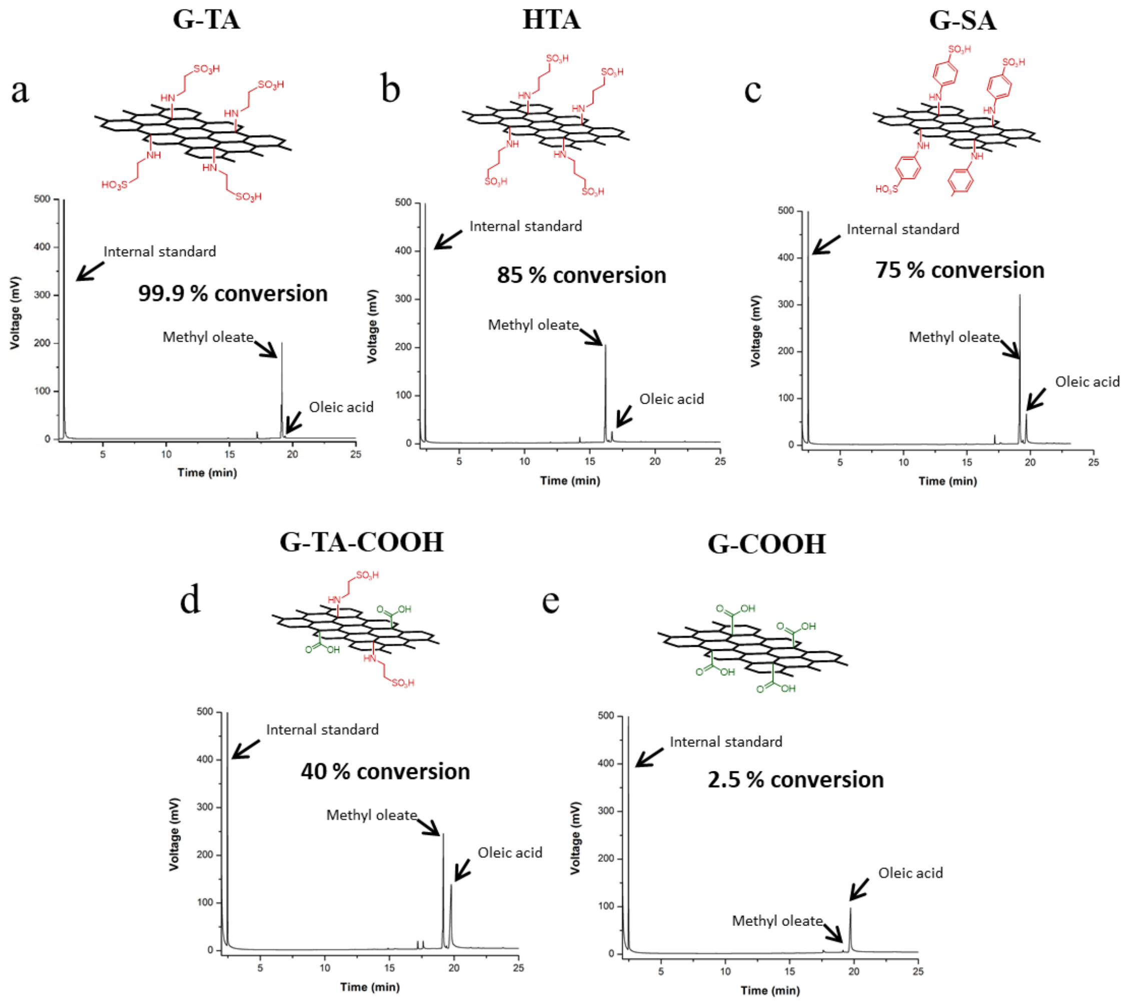

Figure S8. GC graphs of the product after the esterification using graphene functionalized with (a) taurine, (b) homotauine, (c) sulfanilic acid, (d) taurine with carboxylic acid, and (e) carboxylic acid. A schematic representation of each catalyst is shown above the corresponding GC graph.

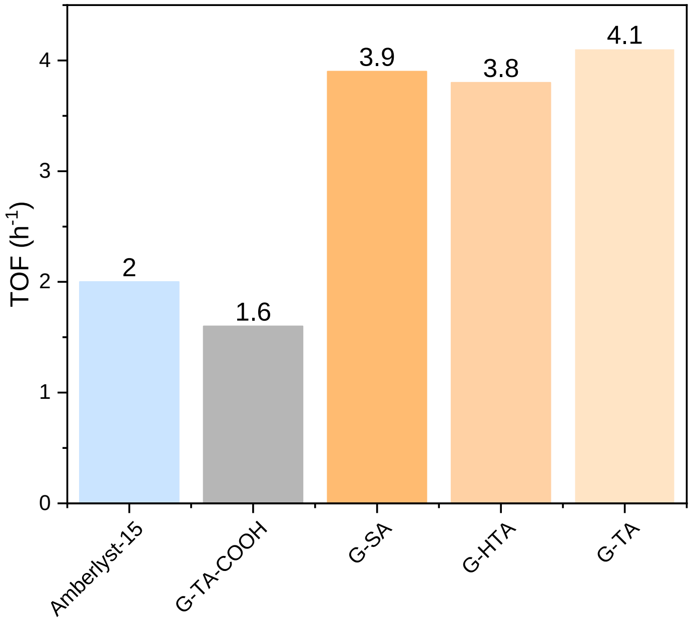

Figure S9. TOF comparison in esterification of oleic acid with different acid-functionalized graphenes and with amberlyst-15. All reactions were performed with a catalyst loading of 7 mass % with respect to oleic acid, at 60 °C. Reaction conditions: oleic acid = 0.5 mmol, methanol =10 mmol, catalyst = 7 mass % wrt oleic acid, continuous stirring at 60 °C for 4 h.

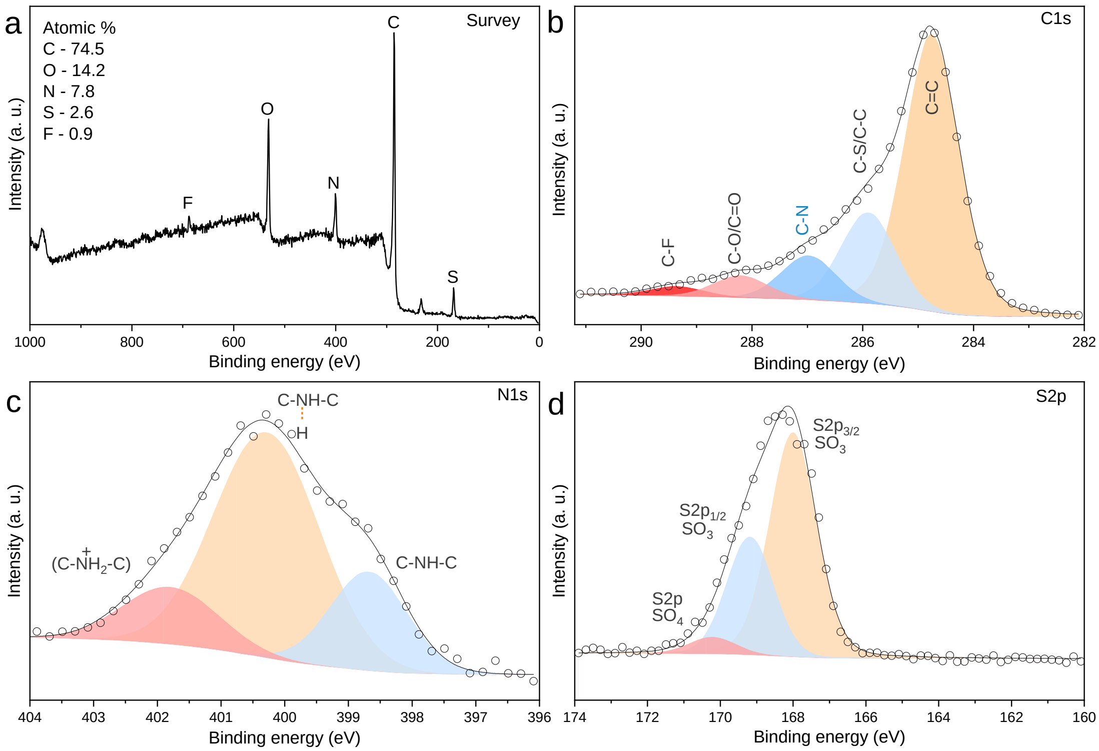

Figure S10. XPS spectrum of G-TA after 5 esterification reactions. (a) survey, core-level, (b) C 1s, (c) N 1s, and (d) S 2p.

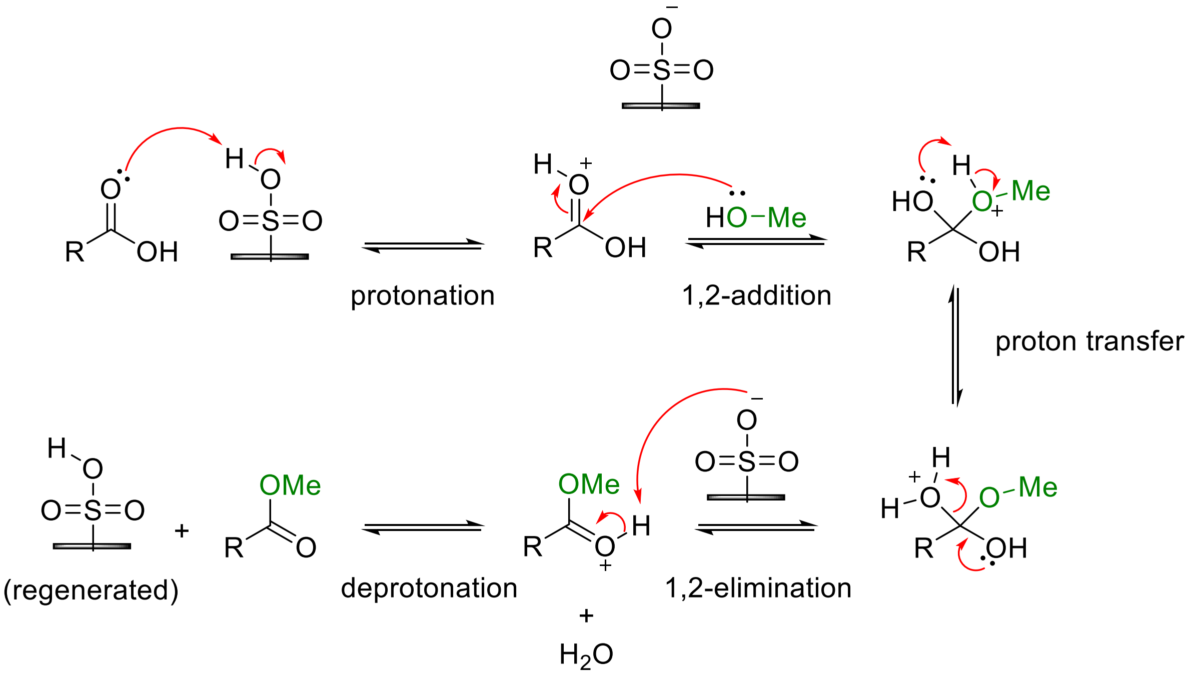

Figure S11. Mechanism for the esterification of fatty acids with methanol.

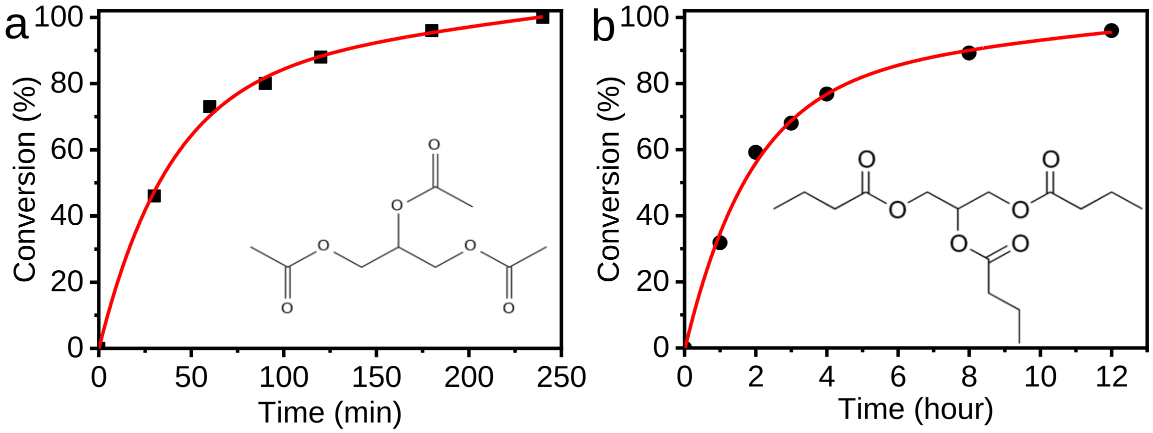

Figure S12. (a) The trans-esterification reaction of triacetin with methanol. (b) Trans-esterification of tributyrin with methanol. Reaction conditions: Triglycerides = 0.5 mmol, methanol = 0.4 mL, catalyst = 7 mass %, with continuous stirring at 60 °C for 4 h.

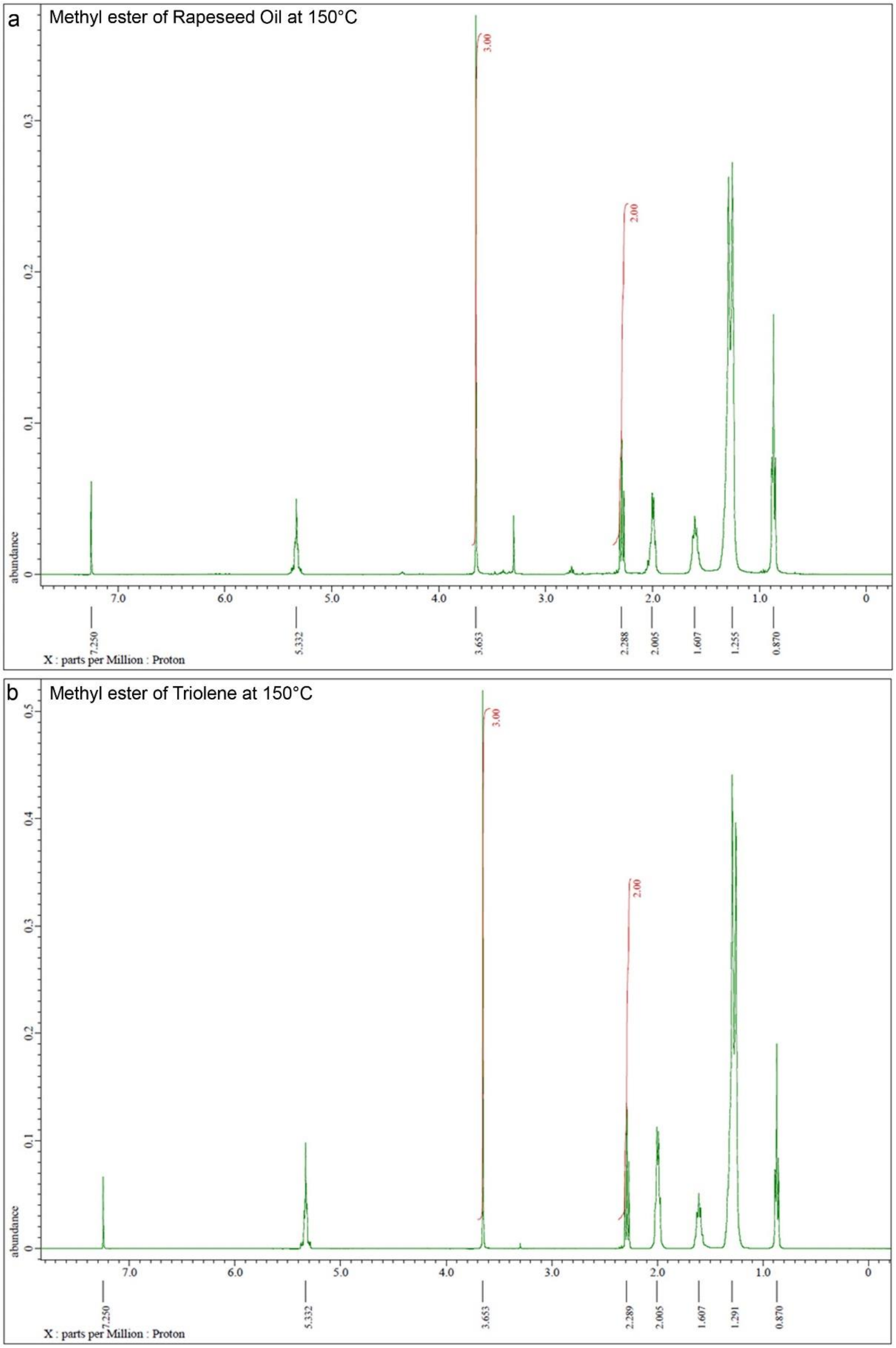

Figure S13. NMR spectrum of methyl esters formed from the trans-esterification of (a) rapeseed oil and (b) triolene.

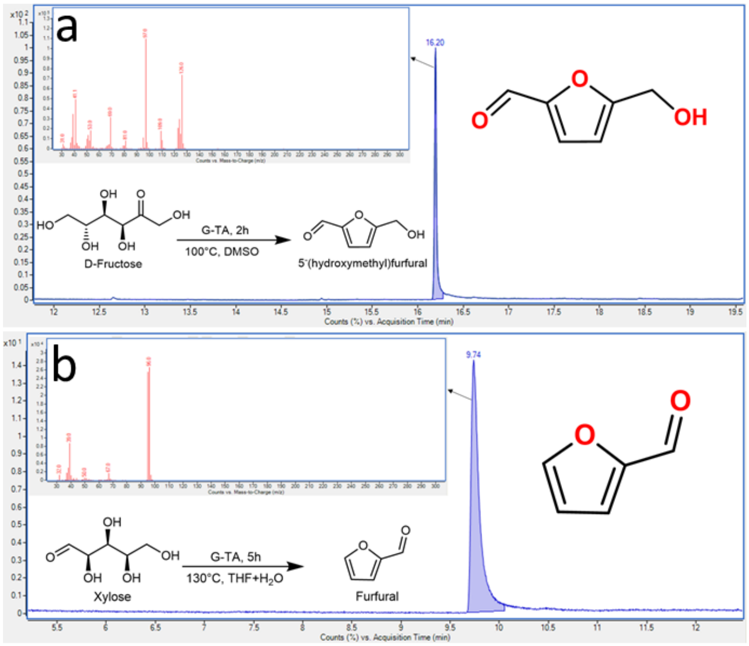

Figure S14. GC-MS spectrum of (a) fructose dehydrated product and (b) xylose dehydrated product.

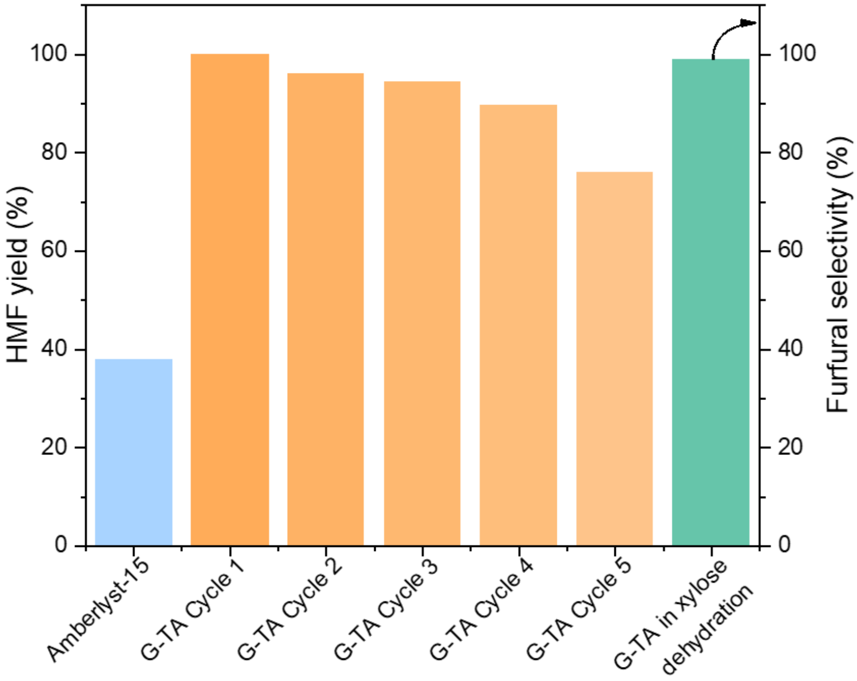

Figure S15. Recycling performance of the G-TA catalyst and the amberlyst-15 for the fructose dehydration reaction. Reaction conditions: fructose = 1 mmol, DMSO = 3mL, Catalyst = 12.5 mg with continuous stirring at 100 °C for 2 h. Green bar represents the xylose dehydration reaction with G-TA catalyst. Reaction conditions: xylose = 2 mmol, Water/THF = 3mL, Catalyst = 25 mg with continuous stirring at 130 °C for 5 h.

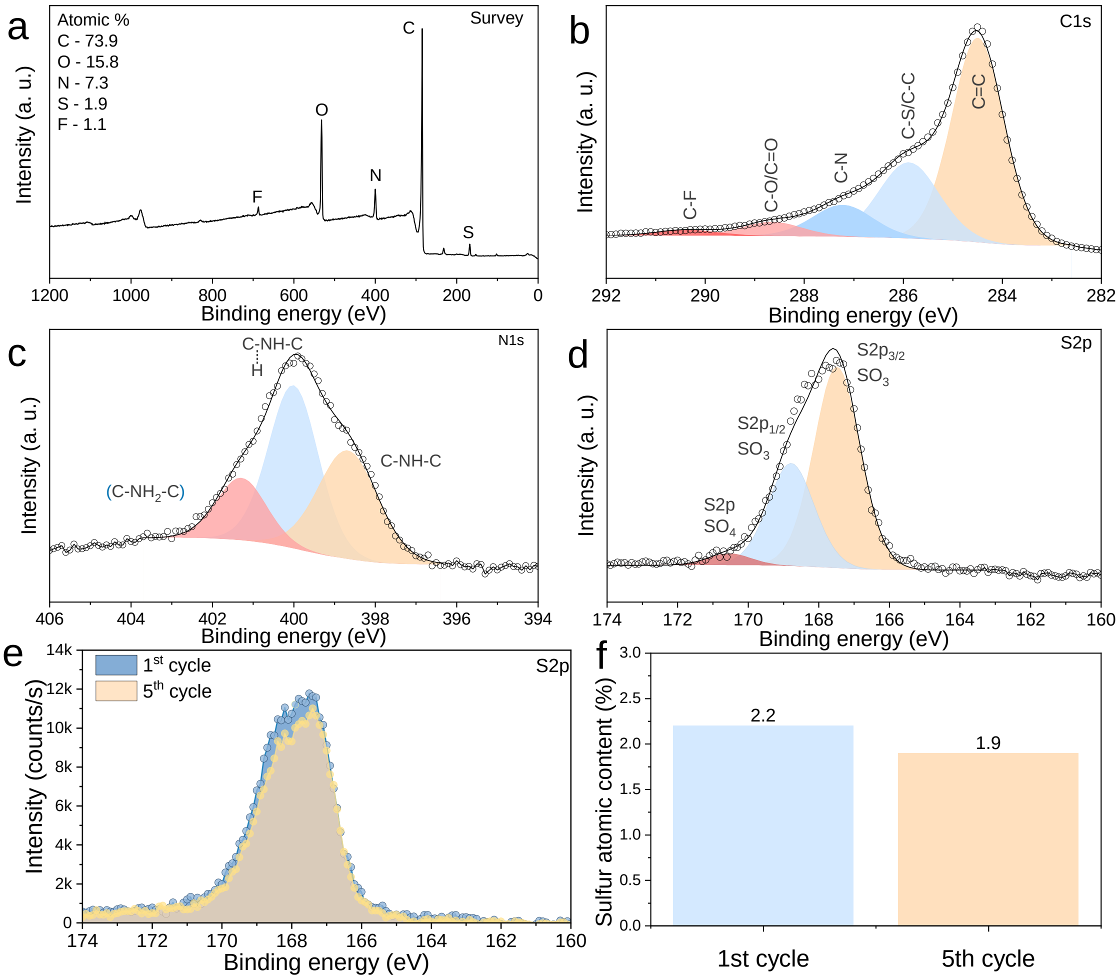

Figure S16. XPS spectra of G-TA after 5 fructose dehydration reactions. (a) Survey, and high-resolution core-level, (b) C 1s, (c) N 1s, (d) S 2p, (e) comparison of the XPS S2p peak intensity of the 1st cycle and the 5th cycle and (f) corresponding sulfur atomic content.

References

[1] A. K. K. Padinjareveetil, M. Pykal, A. Bakandritsos, R. Zboril, M. Otyepka, M. Pumera, *Advanced Science* **2024**, *11*.

[2] M. Toda, A. Takagaki, M. Okamura, J. N. Kondo, S. Hayashi, K. Domen, M. Hara, *Nature* **2005**, *438*, 178-178.

[3] K. Nakajima, M. Hara, *ACS Catalysis* **2012**, *2*, 1296-1304.

[4] X. M. Zhang, Y. P. Zhao, S. T. Xu, Y. Yang, J. Liu, Y. X. Wei, Q. H. Yang, *Nature Communications* **2014**, *5*.

[5] R. Jia, J. W. Ren, X. H. Liu, G. Z. Lu, Y. Q. Wang, *Journal of Materials Chemistry A* **2014**, *2(29)*, 11195-11201.

[6] Y. Wang, D. Wang, M. H. Tan, B. Jiang, J. T. Zheng, N. Tsubaki, M. B. Wu, *ACS Applied Materials & Interfaces* **2015**, *7(48)*, 26767-26775.

[7] I. Ogino, Y. Suzuki, S. R. Mukai, *ACS Catalysis* **2015**, *5(8)*, 4951-4958.

[8] K. Fukuhara, K. Nakajima, M. Kitano, S. Hayashi, M. Hara, *Chemcatchem* **2015**, *7(23)*, 3945-3950.

[9] Y. W. Liu, S. M. Liu, D. F. He, N. Li, Y. J. Ji, Z. P. Zheng, F. Luo, S. X. Liu, Z. Shi, C. W. Hu, *Journal of the American Chemical Society* **2015**, *137(39)*, 12697-12703.

[10] R. B. N. Baig, S. Verma, M. N. Nadagouda, R. S. Varma, *Scientific Reports* **2016**, *6*, 39387.

[11] H. Liu, J. Z. Chen, L. M. Chen, Y. S. Xu, X. H. Guo, D. Y. Fang, *ACS Sustainable Chemistry & Engineering* **2016**, *4(6)*, 3140-3150.
[12] H. M. A. Hassan, M. A. Betiha, S. K. Mohamed, E. A. El-Sharkawy, E. A. Ahmed, *Applied Surface Science* **2017**, *412*, 394-404.
[13] H. L. Zhang, X. Luo, K. Q. Shi, T. Wu, F. He, S. B. Zhou, G. Z. Chen, C. Peng, *Chemsuschem* **2017**, *10*, 3352-3357.
[14] F. S. Liu, X. L. Ma, H. Li, Y. Y. Wang, P. Cui, M. Guo, H. L. Yaxin, W. P. Lu, S. J. Zhou, M. Z. Yu, *Fuel* **2020**, *266*, 117149.
[15] L. Peng, A. Philippaerts, X. X. Ke, J. Van Noyen, F. De Clippel, G. Van Tendeloo, P. A. Jacobs, B. F. Sels, *Catalysis Today* **2010**, *150(1-2)*, 140-146.
[16] L. Geng, Y. Wang, G. Yu, Y. X. Zhu, *Catalysis Communications* **2011**, *13(1)*, 26-30.
[17] K. P. Flores, J. L. O. Omega, L. K. Cabatingan, A. W. Go, R. C. Agapay, Y. H. Ju, *Renewable Energy* **2019**, *130*, 510-523.
[18] G. G. Millán, J. Phiri, M. Mäkelä, T. Maloney, A. M. Balu, A. Pineda, J. Llorca, H. Sixta, *Applied Catalysis a-General* **2019**, *585*.
[19] Y. Wang, T. Len, Y. Huang, A. D. Taboada, A. N. Boa, C. Ceballos, F. Delbecq, G. Mackenzie, C. Len, *ACS Sustainable Chemistry & Engineering* **2017**, *5(1)*, 392-398.
[20] Y. Wang, F. Delbecq, W. Kwapinski, C. Len, *Molecular Catalysis* **2017**, *438*, 167-172.
[21] I. Fúnez-Núñez, C. García-Sancho, J. A. Cecilia, R. Moreno-Tost, E. Pérez-Inestrosa, L. Serrano-Cantador, P. Maireles-Torres, *Applied Catalysis a-General* **2019**, *585*.
[22] R. K. Mishra, V. B. Kumar, A. Victor, I. N. Pulidindi, A. Gedanken, *Ultrasonics Sonochemistry* **2019**, *56*, 55-62.
[23] S. Kumar, M. B. Gawande, J. Kopp, S. Kment, R. S. Varma, R. Zbořil, *ChemSusChem* **2020**, *13*, 5231-5238.
[24] X. Yu, L. Peng, X. Gao, L. He, K. Chen, *RSC Advances* **2018**, *8*, 15762-15772.
[25] B. Karimi, H. M. Mirzaei, H. Behzadnia, H. Vali, *ACS Applied Materials & Interfaces* **2015**, *7*, 19050-19059.
[26] R. Liu, J. Chen, X. Huang, L. Chen, L. Ma, X. Li, *Green Chemistry* **2013**, *15*, 2895-2903.
[27] S. Verma, R. B. N. Baig, M. N. Nadagouda, C. Len, R. S. Varma, *Green Chemistry* **2017**, *19(1)*, 164-168.
[28] X. Qi, H. Guo, L. Li, R. L. Smith Jr., *ChemSusChem* **2012**, *5*, 2215-2220.
